# Clinically relevant small-molecule promotes nerve repair and visual function recovery

**DOI:** 10.1038/s41536-022-00233-8

**Published:** 2022-10-01

**Authors:** Ngan Pan Bennett Au, Gajendra Kumar, Pallavi Asthana, Fuying Gao, Riki Kawaguchi, Raymond Chuen Chung Chang, Kwok Fai So, Yang Hu, Daniel H. Geschwind, Giovanni Coppola, Chi Him Eddie Ma

**Affiliations:** 1 Department of Neuroscience, City University of Hong Kong, Tat Chee Avenue, Kowloon Tong, Hong Kong SAR; 2 Department of Psychiatry, Semel Institute for Neuroscience and Human Behavior, David Geffen School of Medicine, University of California Los Angeles, Los Angeles, CA 90095 USA; 3 Laboratory of Neurodegenerative Diseases, School of Biomedical Sciences, LKS Faculty of Medicine, The University of Hong Kong, Pokfulam, Hong Kong SAR; 4 State Key Laboratory of Brain and Cognitive Sciences, The University of Hong Kong, Pokfulam, Hong Kong SAR; 5 Department of Ophthalmology, The University of Hong Kong, Pokfulam, Hong Kong; 6 Guangdong-Hong Kong-Macau Institute of CNS Regeneration, Jinan University, Guangzhou, China; 7 Department of Ophthalmology, Stanford University School of Medicine, Palo Alto, USA; 8 Program in Neurogenetics, Department of Neurology, David Geffen School of Medicine, University of California, Los Angeles, Los Angeles, CA 90095 USA; 9 Department of Human Genetics, University of California, Los Angeles, Los Angeles, CA 90095 USA

**Keywords:** Regeneration and repair in the nervous system, Neurological disorders

## Abstract

Adult mammalian injured axons regenerate over short-distance in the peripheral nervous system (PNS) while the axons in the central nervous system (CNS) are unable to regrow after injury. Here, we demonstrated that *Lycium barbarum* polysaccharides (LBP), purified from Wolfberry, accelerated long-distance axon regeneration after severe peripheral nerve injury (PNI) and optic nerve crush (ONC). LBP not only promoted intrinsic growth capacity of injured neurons and function recovery after severe PNI, but also induced robust retinal ganglion cell (RGC) survival and axon regeneration after ONC. By using LBP gene expression profile signatures to query a Connectivity map database, we identified a Food and Drug Administration (FDA)-approved small-molecule glycopyrrolate, which promoted PNS axon regeneration, RGC survival and sustained CNS axon regeneration, increased neural firing in the superior colliculus, and enhanced visual target re-innervations by regenerating RGC axons leading to a partial restoration of visual function after ONC. Our study provides insights into repurposing of FDA-approved small molecule for nerve repair and function recovery.

## Introduction

Progress has been made in modifying the hostile central nervous system (CNS) microenvironment by neutralizing inhibitory molecules^1–3^ and enhancing intrinsic growth capacity by introducing foreign genes through viral vector to facilitate axon regeneration^4–12^. Knowledge of the major CNS inhibitors has increased remarkably over the past decades. Much effort has been made to neutralize these inhibitory molecules such as Nogo, myelin-associated glycoprotein and oligodendrocyte-myelin glycoprotein^13,14^. The loss of intrinsic growth capacity of injured CNS neurons represents a major obstacle to successful axon regeneration. It is widely believed that the peripheral nervous system (PNS) regenerates successfully in contrast to the CNS^15–22^; however, which is not entirely true. Damage to the peripheral nerve is followed by a slow rate of axonal regrowth (axons make up peripheral nerve, grow 1 mm/day in rodent/human), and limited function recovery^15,16,22^. The most common type of proximal peripheral nerve injury (PNI) in human (i.e. brachial plexus nerve) and those that involve complete transection of peripheral nerve require long-distance axon regeneration to re-innervate their target muscles. By the time regenerating axons arrive at the distal innervated target muscle whereas muscle atrophy and joint contracture occurs, patients generally have a very limited clinically function recovery even after surgical repair^15,16^. For instance, patients with proximal PNI such as carpal tunnel syndrome and cubital tunnel syndrome (CuTS) who undergo surgery and regain a certain degree of sensory function. However, motor function recovery is extremely limited which depends on time period between the onset of symptoms and surgery termed “critical period”. Our studies showed that there is a limited time window (critical period) in which regenerating axons must reach distal muscle to reform functional neuromuscular junction (NMJ). In mice, the critical period is about 35 days and patient with CuTS (proximal PNI) is 10 months^15,22^.

Genetic modification (gene therapy) that involves introducing regeneration-associated genes (RAGs) on a viral vector into patients with nervous system injuries could offer great hope for cure. While the concept of gene therapy is straightforward, routine clinical implementation needs further development of efficient methods to deliver transgenes specifically to target tissues with high transduction efficiency^23^. There is still much to learn before it becomes a routine, effective and safe medical treatment. Genetic modification might possess undesirable side effects on patients such as causing uncontrolled cell growth resulting in potential malignancy. Therefore, there is an urge to identify small molecules (ideally FDA-approved) as a ready-to-use therapy that could orchestrate multiple signaling pathways required to “switch on” intrinsic growth program of injured neurons for treating nervous system injuries.

*Lycium barbarum* also known as *Fructus Lycii*, Gouqizi or Wolfberry. It is a well-known traditional Chinese medicine and one of the most commonly used Chinese cuisine ingredients. It has been well documented that *Lycium barbarum* polysaccharides (LBP), a major constitute (20–30% by dry mass) and active compound of Wolfberry, exhibits a broad range of beneficial effects. The neuroprotective effects of LBP in neurodegenerative diseases such as glaucoma, Alzheimer’s disease (AD), retinal ischemia and ischemic brain injury have been widely reported^24–26^. Ocular hypertension is one of the major factors leading to glaucoma, a neurodegenerative disease with progressive loss of retinal ganglion cells (RGCs) and optic nerve atrophy. LBP has been shown to promote RGC survival in chronic and acute ocular hypertension model of glaucoma^26,27^. Mice pre-treated with LBP (1 mg/kg) for 7 days effectively protected from ischemic brain damage with a significant reduction in cerebral edema and apoptotic neurons in an experimental stroke model^28^. Accumulation of β-amyloid (Aβ), elevation of glutamine and homocysteine levels are suggested as potential factors contribute to the pathogenesis of AD. Our studies showed that LBP protected cortical neurons against Aβ-induced apoptosis by inhibition of caspases-2 and −3, reduced phosphorylation of RNA-dependent serine/threonine kinase and c-Jun N-terminal kinase^29,30^. LBP also exerted neuroprotective effects on cortical neurons when exposed to high levels of glutamate and homocysteine mediated through the caspase and JNK signaling pathways^31,32^.

In the current study, we explore the clinical relevance of LBP-induced intrinsic growth capacity by microarray and FDA-approved small molecules bioinformatics analysis. We first demonstrated that LBP-induced extensive neurite outgrowth from axotomized dorsal root ganglion (DRG) neurons in the PNS. We then extended these in vitro findings to in vivo studies of functional recovery after severe PNI (mouse model of critical period). LBP not only accelerated function recovery after a single peripheral nerve (sciatic nerve) crush, but also overcame the critical period to enhance sensory and motor function recovery significantly after prolonged muscle denervation^22,33,34^. In the mammalian CNS, axon regeneration in adult mice was undetectable and the loss of retinal ganglion cells (RGCs) was more than 70% at 2 weeks post optic nerve crush (ONC)^35^. Strikingly, LBP promoted RGC survival by 46% and induced robust axon regeneration after ONC. We then explored the clinical relevance of LBP-induced intrinsic growth capacity by performing a large-scale small molecules screening using LBP gene expression profile signature to query a Connectivity map database. The Connectivity map database consists of 1.5 million gene expression profiles generated from cultured human cell lines after treating with ~5000 FDA-approved small molecules where ~1600 of them are involved in Phase 1–3 clinical trials^36,37^. A recent study demonstrated successful identification of small molecules to treat Ewing sarcoma and cervical cancer using this Connectivity map database bioinformatics screening method^38^. We identified two top-ranked FDA-approved small molecules, glycopyrrolate and mexiletine, induced robust axon regeneration and function recovery after PNI. Glycopyrrolate is particularly effective in promoting long-distance optic nerve regeneration, visual target re-innervations and partial visual function recovery after ONC. Current study provides insight into the development of novel therapeutic opportunities direct at enhancing intrinsic growth capacity of injured neurons for PNS and CNS injuries, since repurposing of FDA-approved drugs is time- and cost-saving.

## Results

### LBP promotes the intrinsic growth capacity of injured adult neurons and function recovery after PNI

**Fig. 1:**
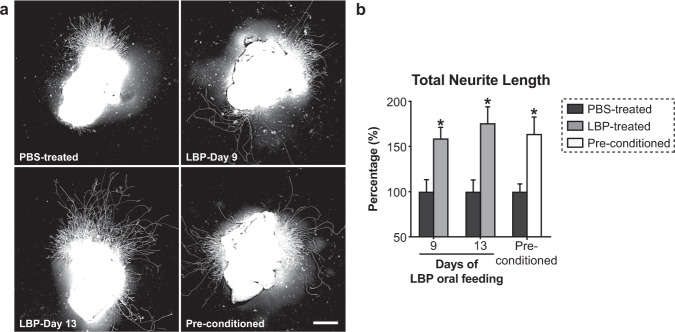
LBP induces intrinsic growth capacity of axotomized dorsal root ganglion (DRG) neurons in ex vivo explant cultures. Adult male C57BL/6 mice were orally administrated with 100 mg/kg LBP (or PBS as vehicle controls) for 9 and 13 consecutive days. For pre-conditioning DRG explant cultures, the left sciatic nerve was transected, and lumbar 3, 4, and 5 (L3/4/5) ipsilateral DRGs were harvested at 5 days post-injury, and cultured on a poly-D-lysine-coated 8-well chamber with a thin layer of Matrigel for 48 h. Cultures were fixed and immunostained with anti-βIII-tubulin antibodies for neurite outgrowth assay. **a** Representative fluorescent micrographs showed that DRG explants treated with LBP for 9 and 13 days induced robust neurite extension compared to the PBS-treated controls. Scale bar: 500μm. **b** The average total neurite length was quantified by automated WIS-Neuromath software. LBP increased neurite outgrowth of DRG explants significantly, with its maximal effect at day 13. The total neurite length of LBP-treated DRG explants (13-day treatment) was comparable with pre-conditioned DRG explants. Mean ± SEM (*n* = 6–8 per group); *P < 0.05, one-way ANOVA followed by post hoc Bonferroni test.

The main purpose of this study is to facilitate clinical application of LBP in nerve repair since it is impractical at large-scale production of LBP that requires tones of wolfberry, and more importantly, the purity of LBP is likely to be affected by the method of extraction and source of wolfberry. We therefore first performed a proof-of-concept experiment to examine the promoting effects of LBP on peripheral nerve repair. Strikingly, LBP induced a marked increase of neurite outgrowth from axotomized neurons in ex vivo DRG explant cultures by 75.8% after oral feeding mice with LBP for 13 consecutive days (Fig. 1a). This is an important proof-of-concept experiment to show that LBP increases the intrinsic growth capacity of neuron, to an extent comparable to that produced by a pre-conditioning peripheral nerve lesion (Fig. 1b)^15^, without eliciting inflammatory responses in neurons^39–41^. The gene expression of pro-inflammatory cytokines (M1 macrophage marker genes), anti-inflammatory cytokines (M2 macrophage marker genes), and chemokines remained unchanged in DRGs after oral administration of LBP for 13 consecutive days (Supplementary Fig. 1).

**Fig. 2:**
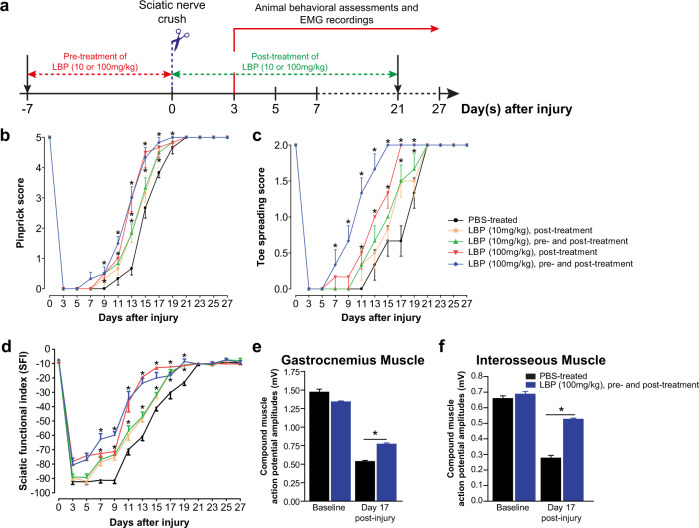
LBP accelerates function recovery after a single sciatic nerve crush (SNC) injury in a dose-dependent manner. **a** Schematic diagram illustrating the experimental timeline for SNC injury and LBP (10 or 100 mg/kg) treatment paradigm. Adult mice received LBP orally once daily for 7 consecutive days (pre-treatment) and 21 consecutive days (post-treatment) immediately after SNC. **b** The beneficial effect of 10 mg/kg and 100 mg/kg LBP was enhanced slightly in mice with 7-day pre-treatment and 21-day post-treatment, when compared with 21-day post-treatment alone. **c** LBP-treated mice (100 mg/kg, pre- and post-treatment) showed the fastest recovery of toe spreading reflexes from days 7 to 19 post-injury when compared with other treatment groups. **d** Gait movement as assessed by sciatic function index, was markedly improved in LBP-treated mice, when compared with PBS-treated controls. **e, f** Improved compound muscle action potential amplitudes of both proximal (gastrocnemius; **e**) and distal (interosseous; **f**) muscles were observed in LBP-treated mice (100 mg/kg pre- and post-treatment) at day 17 post-injury, when compared with PBS-treated controls. Mean ± SEM (*n* = 5–6 per group); *P < 0.05, two-way repeated measures ANOVA followed by post hoc Bonferroni test.

Next, we extended the axon regeneration promoting effect to in vivo studies of function recovery after prolong target muscle denervation using a mouse model of severe PNI. We established a mouse model to delay regenerating axons from reaching distal muscle for 37 days by performing multiple (repeated) sciatic nerve crushes (SNCs). Adult mice usually take 3–4 weeks to regain full function after a single crush^15,16,22,33,34,42^; however, motor function recovery is virtually non-existent if regenerating axons arrived at the motor endplate after 37 days (critical period)^15,22^. We first optimized the therapeutic dosage regime of LBP after a single SNC by animal behavioral tests. LBP accelerated sensory and motor function recovery after a single SNC in a dose-dependent manner. The beneficial effect of 10 mg/kg and 100 mg/kg LBP was enhanced slightly in mice with 7-day pre-treatment, when compared with 21-day post-treatment alone (Fig. 2a–d). Improved motor function recovery was validated by electromyography (EMG) recordings showing an increase of compound muscle action potential amplitude (CMAP) in proximal (gastrocnemius) and distal plantar (interosseous) muscles of adult mice treated with optimal dose of LBP (100 mg/kg) at day 17 post-injury (Fig. 2e, f). Axon and NMJ quantification at multiple time points after a single crush injury demonstrate accelerated axon regeneration (Supplementary Figs. 2–4) and NMJ re-innervation in the target muscles (Supplementary Fig. 5) of LBP-treated (100 mg/kg) mice at days 9, 13 and 17 post-injury.

### LBP overcomes critical period after a severe PNI and induces robust CNS axon regeneration

**Fig. 3:**
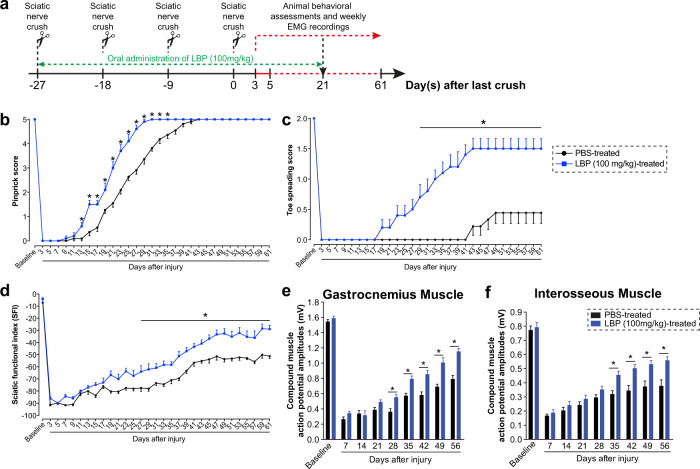
LBP accelerates function recovery after severe peripheral nerve injury and promotes robust CNS axon regeneration. **a** Schematic diagram illustrating the experimental timeline for severe peripheral nerve injury and LBP (100 mg/kg) treatment paradigm. Sciatic nerve was crushed 4 times at 9-day intervals to prevent successful muscle re-innervation for motor function recovery. **b** Pinprick sensitivity was fully recovered at 31 days in LBP-treated mice and 43 days in vehicle control mice. **c** LBP-treated mice were able to regain 75% of the toe spreading reflex, whereas control mice only recovered 22% of toe spreading reflex at 2 months post-injury. **d** Sciatic function index of control mice remained significantly lower than that of LBP-treated mice from day 27 post-injury onwards. **e, f** Functional neuromuscular junction re-innervation was quantified by weekly electromyography recordings of proximal (gastrocnemius) and distal (interosseous) muscles. A significant improvement in the average compound muscle action potential amplitude in LBP-treated mice was observed, reaching up to 68.8% of recovery in the most distal interosseous muscle compared with their own baseline values. Mean ± SEM (*n* = 13 per group); *P < 0.05; two-way ANOVA, followed by post hoc Bonferroni test in (**b**–**f**).

Multiple crush injury is considered to be the most severe form of PNI in mice^15,22^, we crushed the sciatic nerves 4 times at 9-day intervals to prevent muscle re-innervation for 37 days, which missed the critical period for successful motor function recovery. We administrated 100 mg/kg LBP orally immediately after the first crush (27 days) and an additional 21 days after the last crush (Fig. 3a). Sensory function recovery as assessed by pinprick sensitivity, was fully recovered at 31 days in LBP-treated mice and 43 days in vehicle control mice (Fig. 3b). Strikingly, toe spreading reflex in injured hindlimb was regained in LBP-treated mice by 75%. Initial toe spreading response was recorded at 17 days in LBP-treated mice, when compared with first response on day 43 in control mice. Control mice only recovered 22% of toe spreading reflex at 2 months post-injury (Fig. 3c). Quantitative analysis of hindlimb footprints demonstrated a similar improvement of motor function as measured by sciatic function index (SFI). LBP-treated and control mice demonstrated gradually return of motor function over time while SFI of control mice remained significantly lower than that of LBP-treated mice from day 27 post-injury onwards (Fig. 3d). Two months after the last crush, a significant improvement in the average CMAP amplitudes in LBP-treated mice was observed, reaching up to 68.8% of recovery in the most distal plantar muscle compared with their own baseline values. The CMAP amplitude of control mice remained relatively steady throughout the assessment period, with approximately 45.8% recovery in the plantar muscle (Fig. 3e, f). The average axon number in the proximal and distal sciatic nerves was comparable between LBP-treated and control mice indicating that both treatment groups have a similar extent of regeneration 2-month after the last crush (Supplementary Fig. 6). Consistent with the CMAP results, our histology analysis revealed that muscle re-innervation of control mice was 33.8% lower than the LBP-treated mice whereas nearly half of the NMJs remained denervated in control mice 2-month after the last crush (Supplementary Fig. 7).

**Fig. 4:**
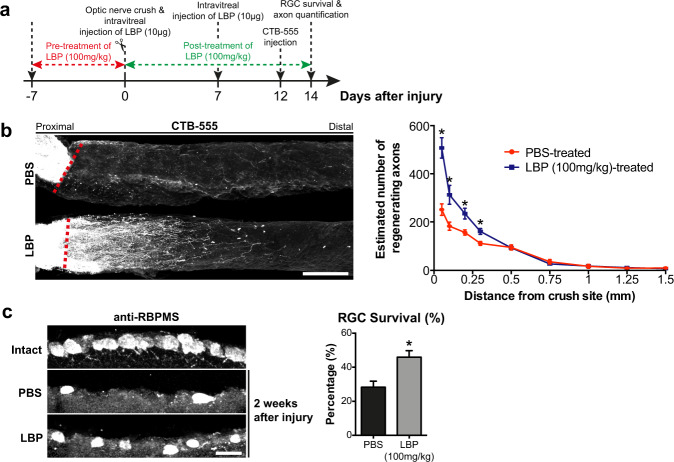
LBP promotes axon regeneration and survival of retinal ganglion cells (RGCs) after optic nerve crush (ONC). **a** Mice received oral administration of LBP (100 mg/kg) for 21 days (7 days pre-treatment and 14 days post-treatment), and intravitreal injections with LBP at days 0 and 7 immediately after optic nerve crush (ONC). **b** Cholera toxin subunit B (CTB)-labeled regenerating axons were quantified in the tissue-cleared optic nerves. LBP-induced robust axon regeneration 2 weeks after ONC. Red dotted line indicated the crushed site. Scale bar: 200 µm. **c** Serial transverse cryosections (20µm-thick) of retinae were immunostained with anti-RBPMS for retinal ganglion cell (RGC) survival assay. RBPMS-positive RGCs were counted in every fifth section per retina (3–5 sections). LBP increased survival by nearly 2-folds, when compared with PBS controls. Scale bar: 20 µm. Mean ± SEM (*n* = 5–6 per group); *P < 0.05; Student’s *t* test in (**b**, **c**).

Given these promising results of LBP in the PNS, we further explored the promoting effect of LBP in the adult mammalian CNS axon regeneration. We administrated LBP orally for 21 days (7 days pre-treatment and 14 days post-treatment) and injected intravitreally with LBP once weekly for 2 weeks immediately after optic nerve crush (ONC) (days 0 and 7 post-crush). On day 12 post-ONC, mice were injected intravitreally with anterogradely transported cholera toxin subunit B (CTB) conjugated with Alexa Fluor 555 (CTB-555) to trace regenerating axons, and the tissue-cleared optic nerves were then imaged with confocal microscope (Fig. 4a)^4,6,12,37,43,44^. Remarkably, LBP increased the number of CTB-labeled regenerating axons substantially (Fig. 4b) and enhanced RGC survival by nearly 2-fold two weeks after ONC (Fig. 4c). Consistent with previous studies demonstrating that intravitreal injection procedure did not trigger neuroinflammation and macrophage infiltration in the retinae^9^, intravitreal injection of LBP did not induce inflammatory cytokine and chemokine gene expression (Supplementary Fig. 8a), and no infiltration of CD68-positive macrophages were observed in the LBP or vehicle-treated retinae (Supplementary Fig. 8b). Our results conclude that LBP induces sustained axon regeneration after PNS and CNS nerve injuries.

### In silico screening of FDA-approved small molecule using LBP gene expression profile signatures

**Fig. 5:**
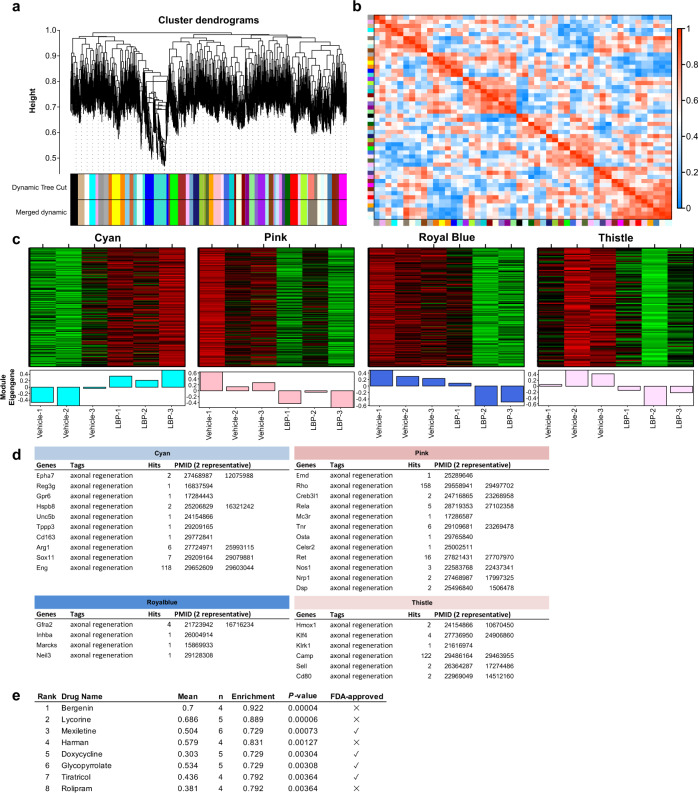
In silico screening of FDA-approved small molecule using LBP gene expression profile signatures. **a**, **b** Weighted gene correlation network analysis identified expression modules across the microarray datasets represented by 50 different colors. Red indicated strong correlations and blue indicated weak or no correlations in (**b**). **c** Four key co-expression modules which were strongly associated with the core signaling network induced by LBP. Heatmap showing the scaled expression of genes (rows) across each sample (vehicle- and LBP-treated dorsal root ganglions) within the four modules. Red indicated up-regulation of genes while green indicated down-regulation of genes. **d** Every single gene within each module was query in PubMed database using keywords such as neuronal regeneration, axon regeneration and nerve injury. LBP-induced expression of RAGs including *Arg1*, *Sox11* and *Tppp3* (in cyan module) and inhibited the expression of growth-inhibitory molecules including *Rho*, *Tnr* (in pink module), and *Klf4* (in thistle module). **e** LBP-induced gene expression profile signatures (both up-regulated and down-regulated) were used to query Connectivity Map database, and eight small molecules (ranked by their permutation *P*-values) with their induced gene expression profiles closely resemble to LBP were identified for subsequent experimental validation based on connectivity and specificity scores.

We then explored the clinical relevance of LBP-induced intrinsic growth capacity by performing a gene expression signature-based in silico small-molecule screening^36,37^. We first performed weighted gene co-expression network analysis (WGCNA) on microarray dataset of DRGs from mice 13 days after oral administration of LBP (showed maximal intrinsic growth in DRGs as shown in Fig. 1), and identified 50 co-expression modules (Fig. 5a, b) (Supplementary Fig. 9). Based on the significance of module-trait relationship (adjusted *P* < 0.05) (Supplementary Fig. 10), we identified 4 key co-expression modules which were strongly associated with the core signaling networks induced by LBP (Fig. 5c). We queried every single gene within each module in PubMed database using keywords such as neuronal regeneration, axon regeneration and nerve injury. LBP-induced expression of RAGs including *Arg1*, *Sox11* and *Tppp3* (in cyan module) and inhibited the expression of growth-inhibitory molecules including *Rho*, *Tnr* (in pink module), and *Klf4* (in thistle module) (Fig. 5d). We then used the LBP-induced gene expression profile signatures to query a public Connectivity Map database. Eight small molecules with their induced gene expression profiles closely resemble to LBP were identified based on connectivity and specificity scores (Fig. 5e).

**Fig. 6:**
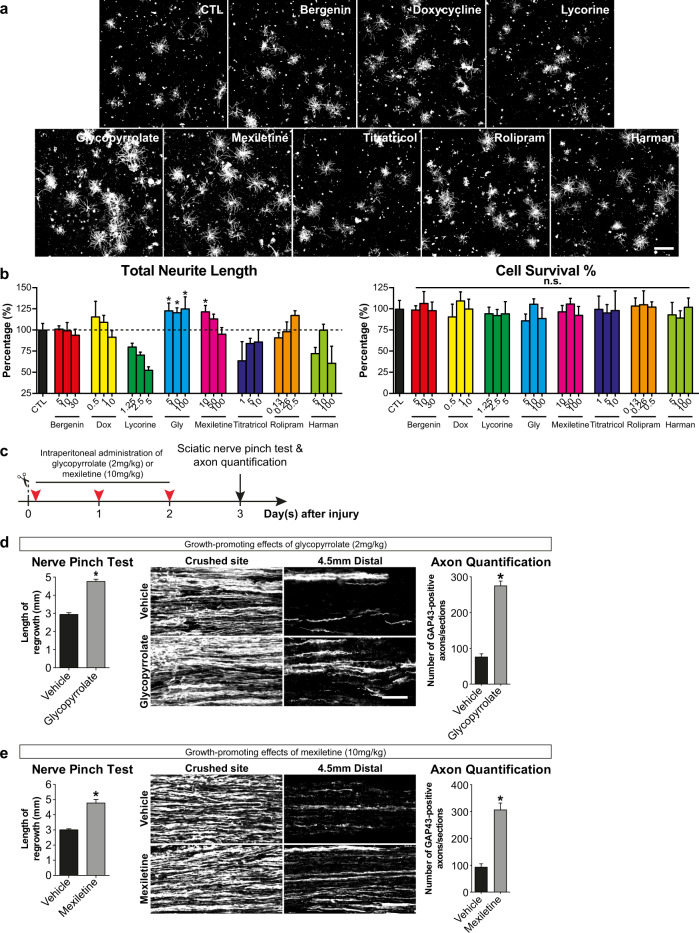
FDA-approved small molecules glycopyrrolate and mexiletine increase the intrinsic growth capacity of axotomized dorsal root ganglion (DRG) neurons, and promote in vivo axon regeneration after sciatic nerve crush injury. **a**, **b** Primary DRG cultures were prepared from adult male C57BL/6 mice, and treated with top-ranked small molecules at various concentrations. DMSO (0.1%) was used as solvent control. Glycopyrrolate and mexiletine induced maximal neurite outgrowth of DRG neurons, when compared with solvent controls. Cell survival assay was performed by WST-1 cell survival assay. All tested small molecules did not exhibit any adverse cell survival effect on DRG neurons. Scale bar: 500 µm. Mean ± SEM of triplicates. **c** Schematic diagram illustrating the experimental paradigm for sciatic nerve pinch test. We performed nerve pinch test in adult mice injected intraperitoneally with glycopyrrolate (2 mg/kg) or mexiletine (10 mg/kg) for 3 consecutive days after injury. **d, e** Both glycopyrrolate (**d**) and mexiletine (**e**) markedly accelerated in vivo axon regeneration after sciatic nerve crush injury as assessed by sciatic nerve pinch tests. The most distal axonal regrowth in mice treated with glycopyrrolate and mexiletine was increased significantly by 61 and 59%, respectively. The number of GAP43-positive regenerating axons were increased by 73% in glycopyrrolate-treated mice and 67% in mexiletine-treated mice. Scale bar: 100 µm. Mean ± SEM (*n* = 5–6 per group); *P < 0.05; one-way ANOVA followed by post hoc Bonferroni test in (**b**); Student’s *t* test in (**d**, **e**).

We validated the promoting effect of eight top-ranked small molecules at various doses and assessed axon regeneration from axotomized DRG neurons in cultures. We demonstrated that two FDA-approved small molecules, glycopyrrolate and mexiletine, induced substantial neurite outgrowth from axotomized adult primary DRG neurons with no adverse effects on cell survival (Fig. 6a, b). The therapeutic potential of glycopyrrolate and mexiletine for PNI were tested in vivo by performing nerve pinch test and histology analysis in adult mice injected intraperitoneally with glycopyrrolate or mexiletine for three consecutive days following SNC (Fig. 6c). In line with our in vitro studies, the most distal axonal regrowth in mice treated with glycopyrrolate or mexiletine was increased by 61 and 59%, respectively. The number of regenerating axons [Growth Associate Protein (GAP)-43 positive] were increased by 73% in glycopyrrolate-treated mice and 67% in mexiletine-treated mice (Fig. 6d, e). Next, to validate the gene expression profiles of LBP-, glycopyrrolate- and mexiletine-treated DRGs, we performed qRT-PCR to verify the expression of the top 10 most significantly differentially expressed genes (DEGs) in mice after LBP, glycopyrrolate or mexiletine treatment (without SNC injury) (Supplementary Table 1). DRGs were harvested from mice that received oral administration of LBP for 13 consecutive days, and intraperitoneal administration of glycopyrrolate or mexiletine for 3 consecutive days. All these treatment paradigms promoted robust intrinsic growth capacity of DRG neurons, as shown in Figs. 1, 6. Of these DEGs, 7 out of 9 genes (excluded one predicted long non-coding RNA gene from the list) were confirmed to be differentially expressed in LBP-treated DRGs, when compared with vehicle controls. Among these 7 DEGs, 6 and 5 genes were differentially expressed in glycopyrrolate-treated DRGs and mexiletine-treated DRGs, respectively (Supplementary Fig. 11). Our results therefore confirmed the therapeutic potential use of both small molecules, which recapitulated the gene signature associated with LBP, in nerve repair.

### Glycopyrrolate induces sustained and long-distance axon regeneration to re-innervate the central visual target areas after ONC

**Fig. 7:**
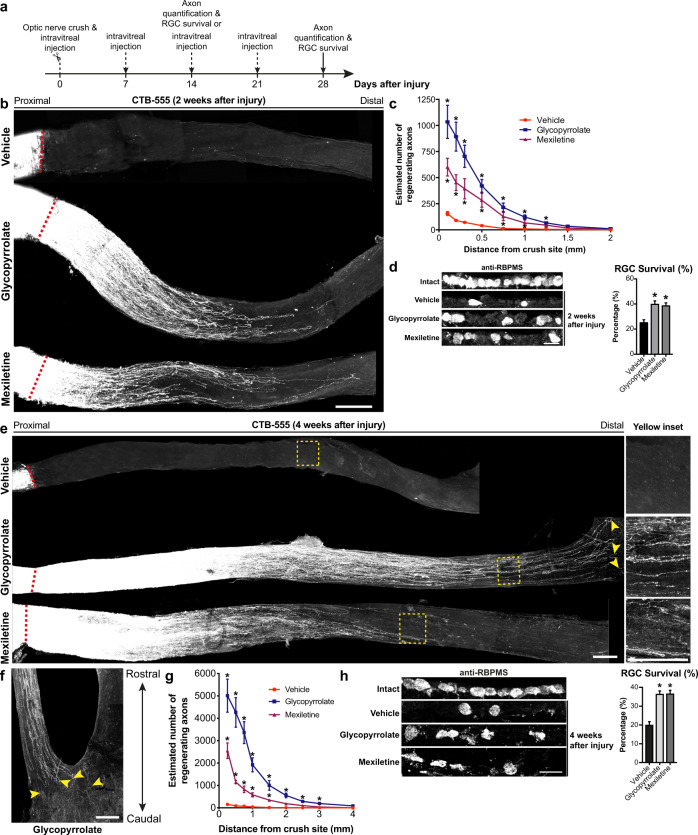
FDA-approved small molecules induce sustained and long-distance axon regeneration after optic nerve crush (ONC). **a** Schematic diagram illustrating the glycopyrrolate (1 µg) and mexiletine (1 µg) treatment paradigm for ONC. Axon regeneration was analyzed by cholera toxin subunit B-555 (CTB-555) tracing. **b** Robust axon regeneration was observed in glycopyrrolate-treated and mexiletine-treated mice at 2 weeks after ONC. **c** Glycopyrrolate and mexiletine induced more than 17-fold and 6-fold increase in the number of CTB-labeled regenerating axons extending 1.0 mm from the site of injury. **d** Serial transverse cryosections (20µm-thick) of retinae were immunostained with anti-RBPMS antibodies for retinal ganglion cell (RGC) survival assay. RBPMS-positive RGCs were counted in every fifth section per retina (3–5 sections). RGC survival rate of small-molecule treatment groups nearly doubled after injury, when compared with vehicle control group. **e** Sustained long-distance axon regeneration was observed in glycopyrrolate-treated mice at 4 weeks after ONC, and some CTB-labeled axons regenerated along the whole length of the optic nerve to reach the optic chiasm in glycopyrrolate-treated mice [yellow insets in (**e**)]. **f** Arrowheads indicated CTB-labeled regenerating axons projecting into the optic chiasm in glycopyrrolate-treated mice at 4 weeks after ONC. **g** At 2 mm distal to the lesion site, glycopyrrolate treatment resulted in more than 70-fold increase in the number of CTB-labeled regenerating axons, while up to 24-fold more regenerating axons were seen in mexiletine treatment group, compared with vehicle controls. **h** At 4 weeks after ONC, both glycopyrrolate and mexiletine induced a similar increase in RGC survival promoting effects, when compared with vehicle-treated controls. Scale bars: 200 µm in (**b**, **e**), 100 µm in (**f**), 20 µm in (**d**, **h**). Mean ± SEM (**n** = 6–8 per group); *P < 0.05; one-way ANOVA followed by post hoc Bonferroni test in (**c**, **d**, **g**, **h**).

To examine the therapeutic potential of glycopyrrolate and mexiletine in CNS axon regeneration, adult mice were injected intravitreally with glycopyrrolate or mexiletine once per week for 2 weeks (days 0 and 7) and 4 weeks (days 0, 7, 14, and 21) as well as daily intraperitoneal injection of glycopyrrolate or mexiletine for 7 consecutive days immediately after ONC (Fig. 7a). At 2 weeks after injury, vehicle control group (saline) showed no regenerating axons beyond the crush site (Fig. 7b) but we observed significant axon regeneration in the glycopyrrolate- and mexiletine-treated mice. Glycopyrrolate and mexiletine induced more than 17-fold and 6-fold increase in the number of CTB-labeled regenerating axons extending 1.0 mm from the site of injury (Fig. 7c). The RGC survival rate of small-molecule treatment groups nearly doubled after injury, when compared with vehicle control group (Fig. 7d). The promoting effect of glycopyrrolate became even more dramatic at 4 weeks after injury, glycopyrrolate triggered intrinsic growth capacity of RGCs that enabled these cells to regenerate axons the entire length of the optic nerve and some of the regenerating axons reaching optic chiasm (Fig. 7e, f). At 2 mm distal to the lesion site, glycopyrrolate treatment resulted in more than 70-fold increase in the number of CTB-labeled regenerating axons, while up to 24-fold more regenerating axons were seen in mexiletine treatment group, compared with vehicle controls (Fig. 7g). Similar survival promoting effect was observed in both glycopyrrolate and mexiletine treatment groups 4 weeks after injury (Fig. 7h).

**Fig. 8:**
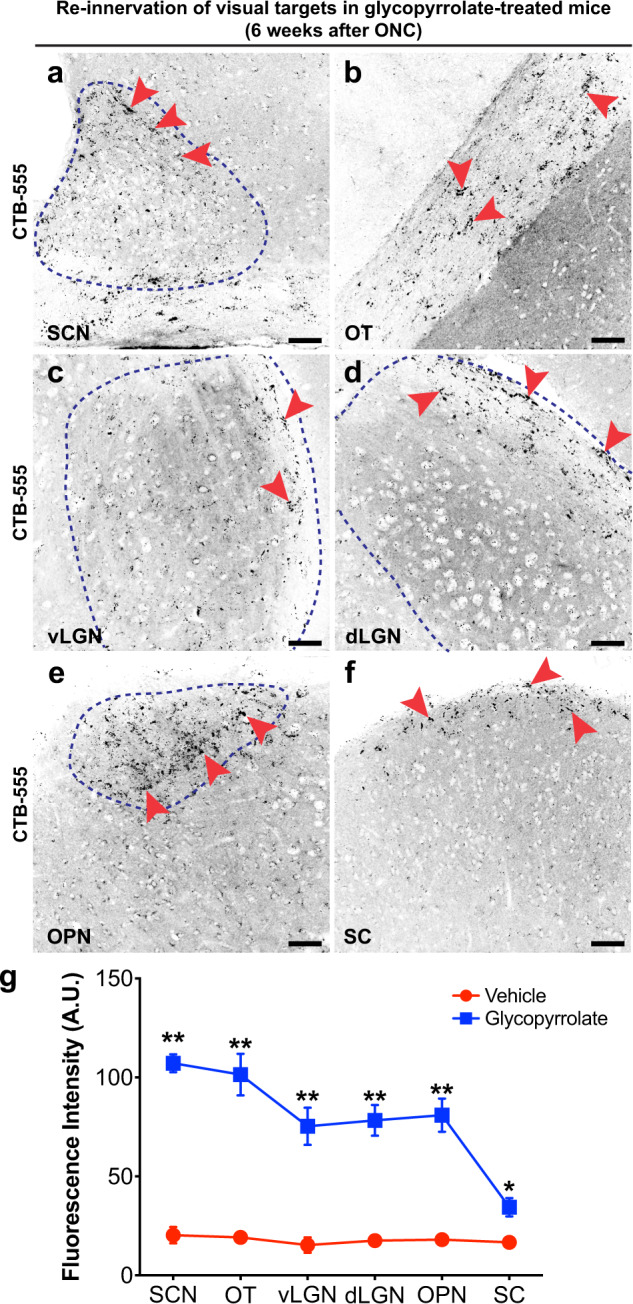
Glycopyrrolate promotes re-innervation at multiple subcortical visual targets. **a**–**f** Representative confocal micrographs demonstrated that a considerable number of CTB-labeled regenerating RGC axons were detected in (**a**) hypothalamic suprachiasmatic nucleus (SCN), (**b**) optic tract (OT), (**c**) thalamic ventral lateral geniculate nucleus (vLGN), (**d**) dorsal lateral geniculate nucleus (dLGN), (**e**) olivary pretectal nucleus (OPN), and (**f**) superior colliculus (SC) in glycopyrrolate-treated mice at 6 weeks post-ONC. Scale bars: 50 µm. **g** The fluorescent intensity of CTB-positive axons in multiple visual targets of glycopyrrolate-treated mice was significantly higher than the vehicle-treated mice. Mean ± SEM (*n* = 3–4 per group). *P < 0.05, **P < 0.01; Student’s *t* test.

To re-establish functional eye-to-brain circuits after ONC, regenerating RGC axons must regrow through the optic chiasm into the optic tract and major visual targets in the brain^45–47^. We questioned whether glycopyrrolate is able to sustain long-distance axon regeneration in the visual system by tracing CTB-labeled regenerating RGC axonal projections at 6 weeks post-ONC. A substantial number of RGC axons were found in the optic chiasm and coursed through the suprachiasmatic nucleus (SCN) in the hypothalamus (Fig. 8a), and spread across the optic tract (OT) (Fig. 8b). The ventral lateral geniculate nucleus (vLGN) (Fig. 8c), dorsal lateral geniculate nucleus (dLGN) (Fig. 8d) in the thalamus, and particular the olivary pretectal nucleus (OPN) in the midbrain (Fig. 8e) were densely innervated. Remarkably, a considerable number of CTB-labeled RGC axons was found at the most distal RGC projection site in the SC (Fig. 8f). The fluorescent intensity of CTB-labeled regenerating RGC axons were quantified in each subcortical visual target area (Fig. 8g). In stark contrast, no CTB-labeled regenerating RGC axons were detected in the subcortical visual targets of vehicle-treated mice (Supplementary Fig. 12).

To ensure that the CTB-positive RGC axons we observed in the optic nerves and major subcortical visual targets were regenerating axons but not spared axons^48^, recombinant CTB conjugated with Alexa Fluor 488 (CTB-488) was intravitreally injected to anterograde label the intact RGC axons two days before the ONC^47^. Immediately after the ONC, glycopyrrolate was intravitreally injected into the injured eye, and CTB-555 was intravitreally injected to trace the regenerating RGC axons one day after the ONC. Optic nerves were harvested three days after ONC and examined histologically (Supplementary Fig. 13a). CTB-488-positive axons were only detected in close proximity to the crush site but none of them regrew beyond the crush site (Supplementary Fig. 13b), suggesting that the ONC procedures were complete with no spared axons. In the same mice, we observed that a number of RGC axons labeled with CTB-555 (injected after ONC) regrew across the crush site after glycopyrrolate treatment at days 3 post-ONC (Supplementary Fig. 13c). In addition, there was no overlapping between CTB-488 (intact uninjured axons) and CTB-555 (regenerating axons) fluorescence in the crushed optic nerve at the distal to the crush site, demonstrating that CTB-labeled axons were regenerating axons but not spared axons.

### Glycopyrrolate treatment elicits neural activity in target brain region and partially restores visual function

**Fig. 9:**
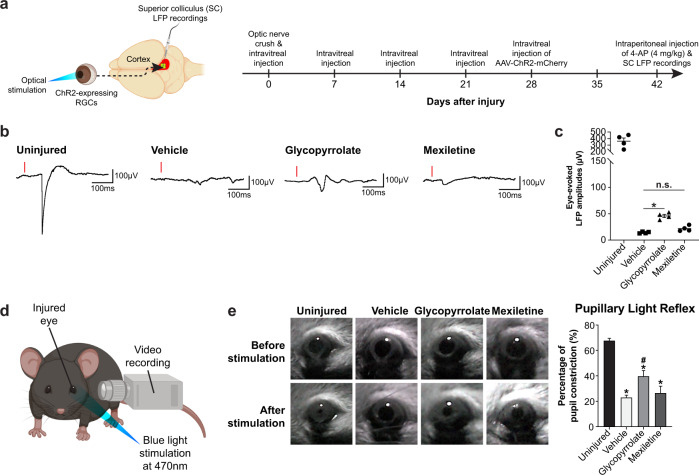
Glycopyrrolate treatment elicits neural activity in target brain region and partially restores visual function. **a** Schematic diagram illustrating the glycopyrrolate or mexiletine treatment paradigm for optic nerve crush (ONC) and superior colliculus local field potential (LFP) recordings upon optical stimulation of retinal ganglion cells (RGCs). **b** Representative eye-evoked LFPs from all treatment groups. Red lines indicate the onset of optogenetic stimulation. **c** We detected minimal eye-evoked LFP from mexiletine-treated mice, as reflected by limited number of regenerating axons in the optic chiasm. In stark contrast, glycopyrrolate treatment markedly increased the maximal eye-evoked LFP by 3.2-fold, when compared with vehicle-treated mice. **d** Schematic diagram illustrating the pupillary light reflex (PLR) test. Mice were allowed to adapt to dark conditions for 1 h before the PLR test. The relative pupil constriction in percentage was calculated as the percentage change in pupil area between the baseline reading after 1-h dark adaptation and 30 s after the light stimulus at 470 nm. **e** Glycopyrrolate treatment partially restored the pupil constriction, while vehicle-treated control mice failed to fully constrict the pupil upon light stimulation at 6 weeks after ONC. Pupillary diameters were comparable in the mexiletine-treated and vehicle-treated mice. Mean ± SEM (*n* = 4 per group in (**c**), *n* = 4–6 per group in (**e**)). *P < 0.05 compared with uninjured eye, ^#^*P* < 0.05 compared with vehicle-treated controls; one-way ANOVA followed by post hoc Bonferroni test. n.s. not significant.

Given that glycopyrrolate treatment induced such a long-distance axon regeneration innervating major visual targets at 6 weeks post-ONC, we questioned whether the regenerated axons are able to restore neural activity and visual function after ONC. We first performed local field potential (LFP) recordings in SC following optogenetic activation of channel rhodopsin-2 (ChR2)-transduced RGCs 6 weeks after ONC. Adeno-associated virus (AAV) encoding channel rhodopsin (ChR2-mCherry) was intravitreally injected into the left eyes 2 weeks before ONC and LFP recordings (Fig. 9a). Consistent with previous study, eye-evoked LFP (359.7 ± 49.9 µV) were recorded from SC successfully in the uninjured eyes by optogenetic activation of ChR2-mCherry-transduced RGCs^49^. As expected, eye-evolved LFP was greatly reduced in vehicle-treated mice (14.3 ± 0.8 µV) at 6 weeks post-ONC. We detected minimal eye-evoked LFP (21.8 ± 2.6 µV) from mexiletine-treated mice, as reflected by the limited number of axons regenerating into the optic chiasm. In stark contrast, glycopyrrolate treatment markedly increased the maximal eye-evoked LFP by 3.2-fold (45.9 ± 3.0 µV), when compared with vehicle-treated mice (Fig. 9b, c). Finally, we performed pupillary light reflex (PLR) test, which is a widely used clinical tool to assess the integrity of visual pathways^50^. Mice were allowed to adapt to dark condition for 1 h and were then presented a short-wavelength (blue) light to the dark-adapted dilated eyes (Fig. 9d). Quantitative analyses were performed by examining the average change in pupil area following light illumination. In the uninjured intact eyes, we detected a 67.9 ± 1.7% reduction in pupil diameter, while the vehicle-treated ONC mice was unable to fully constrict the pupil (23.0 ± 1.8%) upon light stimulation. Glycopyrrolate treatment led to partial restoration of the pupil reflex and visual function (39.9 ± 4.4% reduction in pupil diameter). However, pupillary diameters in mexiletine-treated mice (26.4 ± 5.3% reduction) and vehicle-treated mice were similar after ONC (Fig. 9e). Taken together, our results indicate that sustained long-distance axon regeneration and reformation of functional synapses with their targets in the brain (neural firing) are crucial for successful visual function recovery.

## Discussion

Traumatic injuries to the PNS and CNS are the leading cause of disability and the second leading cause of death worldwide^51^. Nervous system injuries often result in catastrophic loss of motor function, and are indeed the most challenging problems faced by clinicians and research scientists. The first step for successful nervous system regeneration is to accelerate axonal regrowth from injured neurons, which requires activation of intrinsic growth capacity of injured neurons. Optic nerve injury such as traumatic optic neuropathy results in permanent vision loss. Motor vehicle and bicycle accidents, head injuries, falls and contact sports account for the majority of causes^52,53^. Common medical procedures for treating patients with traumatic optic neuropathy include surgical decompression of optic nerves and high-dose corticosteroids (methylprednisolone). However, a large-scale clinical study performed in 16 countries suggest that either alone or in combination of both treatments did not significantly improve the visual acuity in patients^54^. Here, we first demonstrate that LBP exerts exceptional phenotypic benefit in axon regeneration and function recovery. Nevertheless, LBP constitutes only 20–30% of the dry mass of wolfberry, which makes it impractical and costly at large-scale purification. We therefore proposed a bioinformatics platform for mining FDA-approved small molecules by querying a Connectivity map database with gene expression signatures of LBP. Two FDA-approved small molecules, mexiletine and glycopyrrolate, were identified through this “phenotype-based” bioinformatics platform. Mexiletine exerts its therapeutic action through blockade of voltage-gated sodium channels results in the inhibition of inward sodium current required for the initiation of impulses, thus reducing action potential. Mexiletine is a widely used class IB anti-arrhythmic drug to treat irregular heartbeat (arrhythmias), and considered as an oral analogue of lidocaine to provide pain relief in patients with PNI and diabetic neuropathy^55,56^. Mexiletine is a well-known sodium channel blocker and accumulating evidence demonstrate that electrophysiological properties of axonal sodium channels have been linked with axon regeneration^57^. Glycopyrrolate is an antagonist of muscarinic acetylcholine receptor (mAChR), which has been used to treat sialorrhea in children patients with neurological disorders such as cerebral palsy to reduce drooling, and to treat peptic ulcers in adult patients to reduce stomach acid secretion^58,59^. Given the fact that glycopyrrolate is a commonly used non-selective mAChR antagonist, a recent study showed that blockade of M1 mAChR promoted neurite outgrowth of adult sensory neurons in vitro, and protected peripheral nerve terminals from degeneration in rodent models of diabetic neuropathy and chemotherapy-induced peripheral neuropathy^60^. Our experimental validation has confirmed that glycopyrrolate imposed greater promoting effect than mexiletine in mammalian CNS axon regeneration. In fact, elevated level of zinc (Zn^2+^) in injured RGCs contributed to neuronal apoptosis and regeneration failure after ONC^61^. As the uptake of Zn^2+^ was mediated by M1 mAChR^62^, inhibition of Zn^2+^ uptake in injured RGCs by using mAChR antagonists such as glycopyrrolate might facilitate RGC survival and axon regeneration after ONC, given that mAChR were abundantly expressed in the retinae^63^.

In the retina, rod and cone photoreceptors forward visual information via interneurons to RGCs. To facilitate the recovery of visual function after injury, axons of RGCs must regenerate through the optic nerve and relay nerve impulses to proper subcortical visual targets in the brain for image processing and formation^64–66^. RGCs can be classified into 46 distinct subtypes based on their transcriptional profiles^67,68^. The link between RGC subtype specificity and the capacity for axon regeneration has only very recently been demonstrated that requires further investigation^68–70^. For instance, injured axons from intrinsically photosensitive RGCs (ipRGCs), a subset of RGCs that express photopigment protein melanopsin, must regenerate and re-innervate to OPN in order to restore PLR^64,66^. ipRGCs usually survive well whereas subtypes such as ON–OFF direction-selective RGCs are completely lost after ONC^69,71,72^, possibly due to the fact that ipRGCs are able to maintain a high mTOR activity after ONC^73^. In the current study, we showed that glycopyrrolate treatment sustained long-distance axon regeneration along the entire length of the optic nerve, reaching the optic chiasm at 4 weeks post-ONC and finally re-innervated multiple visual targets as evidenced by CTB-labeled regenerating RGC axons and increased neural firing at the most distal RGC projection site in the SC upon optogenetic activation of injured RGCs. Glycopyrrolate treatment also showed promising result in partially restoring PLR at 6 weeks post-ONC. Consistent with that reported in previous studies, OPN is one of the retinorecipient nuclei of the pretectum which has been studied extensively as a key structure responsible for the onset of PLR (retino-OPN pathway)^50,66^. Our histological studies demonstrated clear anatomical evidence of regeneration in major visual targets including the OPN (Fig. 8e), supporting the notion that a complete restoration of neural pathways for visual function might require a strong re-innervation by regenerating RGC axons. Nevertheless, to the best of our knowledge, that such a long-distance axon regeneration induced by a single molecule/gene within four weeks after optic nerve injury, has not been reported. Indeed, combination treatments is often required for long-distance RGC axon regeneration. For instance, co-deletion of PTEN and SOCS3, together with CNTF overexpression, leading to sustained axon regeneration and some axons could be found in the SCN^6,74,75^ and form synapses in the SCN^74^. A combination of three treatments including Zymosan, cAMP, and PTEN deletion, resulting in partial visual function recovery detected by visually guided behavior (visual cliff)^46^. In a recent study, long-distance axon regeneration to multiple subcortical visual targets including SCN, thalamic vLGN and dLGN were observed by enhancing RGC neural activity along with the elevated levels of mTOR resulting in partial restoration of visual function^47^. Combinatorial treatment strategies that involve multiple molecules/genes and pathways would certainly be necessary for clinically meaningful regeneration of RGC axons. Further evaluation of glycopyrrolate (alone or in combination with other growth-promoting molecules/genes) in a clinical setting is thus warranted.

In conclusion, the proposed bioinformatics platform proves an effective strategy for finding drug repurposing opportunities and more economically competitive than testing thousands of small molecules by biological assays. Repurposing of existing FDA-approved drugs is time saving that normally Phase I clinical trial can be skipped. Several clinical studies demonstrated the feasibility of non-viral intravitreal administration of drugs and stem cells in patients with neovascular age-related macular degeneration and retinitis pigmentosa^76–78^. These advantages make our bioinformatics platform an appealing tool for lead discovery across disease areas to avoid the need for challenging and labor-intensive identification of functional relevant modulators.

## Methods

### Animals

Adult male C57BL/6J mice (8–12 weeks old) were used for all experiments. All mouse husbandry and euthanasia were performed in compliance with the Institutional Animal Care and Use Committee (IACUC) guidelines. Surgical procedures performed were in accordance with protocols approved by the City University of Hong Kong Animal Research Ethics Sub-Committee and Department of Health. HKSAR.

### LBP administration and treatment paradigm

Brown freeze-dried powder of LBP was freshly dissolved in phosphate buffered saline (PBS) at 10 mg/ml before use^29^. For single sciatic nerve crush studies, adult mice were divided into three groups: oral LBP treatment for 21 consecutive days immediately after the crush (post-treatment group); oral LBP treatment for 7 consecutive days before the crush and oral LBP treatment for 21 consecutive days immediately after the crush (pre- and post-treatment) and vehicle controls (PBS) (Fig. 2a). For multiple sciatic nerve crush studies, we administrated 100 mg/kg LBP orally immediately after the first crush for 27 consecutive days and an additional 21 consecutive days after the last crush (Fig. 3a). For mouse model of optic nerve crush (ONC) injury, adult mice received intravitreal injection of 10 µg LBP (10 µg/µl) at days 0 and 7 post-ONC, and oral administration of LBP for 7 days (pre-treatment) and 14 days (post-treatment) at a dose of 100 mg/kg (Fig. 4a). Mice were weighed every week throughout the course of the study to monitor health condition in general.

### Ex vivo DRG explant cultures and neurite outgrowth assay

Adult mice were treated orally with 100 mg/kg LBP or PBS for 9 or 13 consecutive days. Lumbar 4 and 5 (L4/5) DRGs, which supply the sciatic nerve, were dissected and cleaned of spinal and peripheral roots, and plated onto 8-well glass chamber slide (Millipore) coated with Matrigel (BD Biosciences)^16,79^. DRG explants were cultured in full Neurobasal (NB) medium supplemented with B27, 200mM L-glutamine, 50 ng/ml NGF (Invitrogen), 2 ng/ml GDNF (Sigma-Aldrich) and 10 μM Ara-C. After 48 h of incubation, DRG explants were fixed with 4% paraformaldehyde (PFA) and immunostained with anti-βIII-tubulin antibodies (Sigma-Aldrich) and Alexa Fluor 488-conjugated secondary antibodies (Molecular Probes) for neurite outgrowth assay.

Neurite outgrowth assay was performed to quantify total neurite length in DRG explant cultures^16,79^. For each DRG, non-overlapping quadrant images were taken at 4× magnification using an epifluorescence microscope (Nikon Eclipse Ni-E) equipped with a motorized stage. Total neurite length was quantified using automated WIS-NeuroMath software (Weizmann Institute of Science). Data were obtained from 8–10 DRG explants from at least three independent experiments repeated in triplicate.

### Sciatic nerve crush (SNC) and prolonged target muscle denervation

Sciatic nerve crush (SNC) was performed on deeply anesthetized (under 2.5% isoflurane) adult male C57BL/6 mice (8–12 weeks old)^15,33,34^. Briefly, left sciatic nerve was exposed, separated carefully from surrounding connective tissue and crushed with smooth forceps (Fine Science Tools) for 15 s at the level of external rotator muscles, distal to the sciatic notch. The site of injury was completely flattened and transparent indicating complete crush. For mouse model of prolonged muscle denervation, 4 sciatic nerve crushes were made at 9-day intervals which prevented muscle re-innervation for 37 days (critical period) (Fig. 3a)^15,22^. After the SNC, overlying muscles and skin were sutured and the mice were allowed to recover on heated pads. The surgeons who performed the surgery was blinded to the treatments.

### Sensory and motor function recovery assessments

On day 3 after the last sciatic nerve crush in single or multiple crushed mice, we first performed pinprick assay, and followed by toe spreading test and sciatic function index (SFI) measurement on the same mouse every other day with 30 min apart from each test in a sequential manner^15,22,33,34,80^. Behavioral tests were done blinded to surgery and treatments. Mice were habituated (30 min) for three sessions the week before taking baseline readings and surgery.

Pinprick assay was performed to measure successful regeneration of sensory axons into the hindlimb skin^15,22,33,80^. Insect pin (FST) was applied gently from the most lateral toe (distal) to the heel (proximal) of ipsilateral hindlimb (divided into 5 areas). A response is considered as positive when the mouse withdraws its hindlimb briskly, and the mouse is scored 1 for this area and test for the next one distally until reaching the heel (score 0: no recovery; score 5: full recovery). Each mouse received two rounds of pinprick tests on the same day with 30 min apart to confirm scores.

To assess motor function recovery, toe spreading test and SFI measurement were performed 1 h after the pinprick assay in a sequential manner^15,22,33,80^. Mice were gently covered with a piece of cloth and picked up by the tail. Toe spreading reflex is scored as 0—no spreading; 1—intermediate spreading with all toes separated for less than 2 s; and 2—full spreading with all toes completely and widely spread for at least 2 s. Mice were scored only when a full response is observed on the contralateral side to the injury. Mice were evaluated twice in each experimental session with at least a 30-min interval.

For SFI measurement, mice were trained to walk down a narrow corridor covered with white paper strip (10 × 60 cm) and the hindlimbs were pained with red water color. SFI baseline values were taken after three independent training sessions spanning across the week before injury. SFI was calculated from footprints using the formula as shown below^22,33^:

$$\begin{array}{l}{{{\mathrm{SFI}}}} = - 38.3\left( {{{{\mathrm{EPL}}}}-{{{\mathrm{NPL}}}}} \right)/{{{\mathrm{NPL}}}} + 109.5\left( {{{{\mathrm{ETS}}}}-{{{\mathrm{NTS}}}}} \right)/{{{\mathrm{NTS}}}} \\ \quad\qquad+ \, 13.3\left( {{{{\mathrm{EITS}}}}-{{{\mathrm{NITS}}}}} \right)/{{{\mathrm{NITS}}}} - 8.8\end{array}$$

Print length (PL)—distance from the heel to the third toe; Toe spread (TS)—distance from the first to the fifth toe; Intermediary toe spread (ITS)—distance from the second to the fourth toe; Experimental (E)—Ipsilateral side to injury; Naïve (N)—contralateral side to injury.

Four clear and distinct footprints were taken from both the left ipsilateral hindlimbs and the right contralateral hindlimbs for SFI calculation. Mice with SFI values close to 0 indicates normal gait movement, while the motor function is severely impaired if the SFI values close to −100.

### Electromyography (EMG) recording of gastrocnemius (proximal) and interosseous (most distal) muscles

Mice were anesthetized with ketamine (100 mg/kg)/xylazine (10 mg/kg) for EMG recording^22,33,34,80^. Proximally, the active and passive stimulating electrodes were inserted at the sciatic notch and paravertebrally into the dorsal aspect of the animals (20 mm from active electrode), respectively. Distally, the active and passive stimulating electrodes were inserted subcutaneously at the Achilles tendon and gastrocnemius muscle (20 mm from active electrode), respectively. Compound muscle action potential (CMAPs) of gastrocnemius muscle was recorded by using active electrode inserted into gastrocnemius muscle and Achilles tendon electrode as reference. Proximal stimulation was used for CMAP of gastrocnemius muscle. CMAP of interosseous muscle was recorded by two pin electrodes. The active and reference electrodes were inserted into the first and fourth interosseous muscle of the same paw, respectively. Proximal and distal stimulation were used for CMAP of interosseous muscle. Mean CMAP amplitude was recorded (Blackrock microsystem, USA), and calculated from 5–6 peaks (Spike 2, UK)^22,33,34,80^.

### Axon and neuromuscular junction (NMJ) quantification

To evaluate axon regeneration histologically by the number of neurofilament (NF) labeled axons after SNC^15,22,33,34^. On days 9, 13, 17, anesthetized mice were transcardially perfused with 4% PFA. Sciatic nerve (from 5-mm proximal to the crush site to the level of flexor retinaculum in the ankle, 25 mm in total length), gastrocnemius and interosseous muscles were collected. PFA-fixed sciatic nerve was divided into 5-mm segments, and 4-μm-thick cryosections were immunostained with anti-NF antibodies. Number of anti-NF-labeled axons in proximal 5 mm, distal 5-, 10-, 15-, 20- and 25-mm segments were quantified using ImageJ (NIH).

For NMJ quantification, 20-μm-thick cryosections were immunostained with anti-NF-200 (axon) and anti-α-bungarotoxin (NMJ) antibodies. Re-innervation was quantified for overlapping NF-200 and α-bungarotoxin immunoreactivity. Re-innervation in about 600–800 NMJs were categorized as either innervated (fully overlapped) or denervated (no overlapping) in every fourth section per mouse^15,22,33,34^. Representative photomicrographs at high magnification were imaged using Carl Zeiss LSM 880 confocal microscope equipped with Airyscan Module.

### Microarray analysis

Adult mice were treated orally with 100 mg/kg LBP (or PBS as vehicle control) for 13 consecutive days based on our ex vivo DRG explants cultures to achieve maximal intrinsic growth capacity in DRG neurons. L4/5 DRGs were dissected from 3 separate groups of mice (*n* = 3 mice per group), total RNA was extracted using TRIzol reagent (Invitrogen), and 3 biologically independent microarray analysis were performed. Total RNA concentration was measured on a NanoDrop 8000 Spectrophotometer (Thermo Fisher Scientific), and RNA integrity was assessed using the Bioanalyzer (Agilent). Five hundred nanograms of high-quality total RNA obtained from DRGs of each group was amplified, fragmented, labeled and hybridized to the GeneChip Mouse Gene 2.0 ST Array (Affymetrix).

The microarray data was imported into R software, and pre-processed using the ‘expresso’ function and the MAS5 method^37^. The correlation of gene expression between samples was calculated. Outliers with mean sample correlations more than three standard deviations below average were omitted. To determine differential expression of each candidate gene, we performed quantile normalization on the pre-processed microarray data.

### Weighted gene co-expression network analysis (WGCNA)

WGCNA was performed to identify groups of genes (i.e. modules) that are differentially expressed after LBP treatment in DRG neurons using R package^37^. Briefly, 10,000 most variable genes based on variance/standard deviation were selected to construct signed networks. The Pearson correlations between each pair of selected genes was computed to yield a similarity (correlation) matrix. The adjacency matrix was calculated by raising the absolute value of the co-expression correlation matrix to a power β = 16. Minimum module size was set to 100. The adjacency matrix was then used as a measure of node similarity, based on the topological overlap matrix. Using hierarchical clustering methods, the modules were interconnected based on topological overlap measure and the final modules were determined by merging similar expression profile with cut height threshold (MEDissThres = 0.1)

Consensus module analysis was performed to detect sets of highly connected nodes shared in multiple networks. For module detection, adjacency matrices were transformed into measures of dissimilarity and input as a form of hierarchical clustering. Under this circumstance, modules were defined as cluster of genes with high topological overlap. Based on consensus module analysis, the first principle component of gene expression in each module was calculated (i.e. module eigengene). Finally, modules that were strongly associated with LBP treatment were identified based on the significance of module-trait relationship (i.e. Bonferroni corrected *P*-value < 0.05) (Supplementary Fig. 10).

### Gene ontology (GO), pathway enrichment and PubMed analysis

Gene ontology (GO) and pathway enrichment analysis were performed using Database for Annotation, Visualization and Integrated Discovery (DAVID) platform^37^. Briefly, candidate genes from each module were used for GO and pathway enrichment analyses. Enriched GO terms and pathways with Benjamini corrected P-values less than 0.05 were selected. PubMatrix were used for literature mining of PubMed to identify potential correlation of each module with axon regeneration by testing association with keywords including neuronal regeneration, axon regeneration and nerve injury in the PubMed database for each candidate gene.

### In silico small-molecule screening

The Connectivity map database (build 02) was used for small-molecule screening by evaluating the similarity between query signature (LBP signature) and more than 1.5 million gene expression profiles for over ~5,000 commercially available FDA-approved small molecules^36,37^. Candidate genes with significant differential expression levels (either up- or down-regulated) at *P* < 0.005 were selected as “query signatures (LBP signature) based on the microarray analysis. Based on a non-parametric, rank-based pattern-matching Kolmogorov-Simirno statistics, LBP-induced genes in the expression profile was estimated with a metric to produce a “connectivity score” ranging from +1 (strong correlation) to −1 (strong anti-correlation). The probe ID was defined by Affymetrix Mouse Gene 2.0 ST Array, and those probe IDs corresponding to the LBP signatures were mapped using DAVID and followed by the query in the Connectivity map database. The mean of the connectivity scores, statistical significance of each identified candidate gene (i.e. permutation *P*-value) and the null percentages were used to formulate permutated results, and to rank the small molecules based on their statistical significance of the connectivity scores. The top eight ranked small molecules were selected for experimental validation using in vitro DRG cultures and in vivo sciatic nerve pinch test.

All small molecules were purchased from Selleckchem and dissolved in 0.1% DMSO according to manufacturer’s instruction. We used 0.1% DMSO as vehicle control. Top eight small molecules were selected for DRG neurite outgrowth assays at three different doses, including bergenin (5, 10 and 30 µM), doxycycline (0.5, 1 and 10 µM), lycorine (1.25, 2.5 and 5 µM), glycopyrrolate (5, 10 and 100 µM), mexiletine (10, 50 and 100 µM), titratricol (1, 5 and 10 µM), rolipram (0.13, 0.26 and 0.5 µM) and harman (5, 10 and 100 µM).

### Primary dissociated DRG cultures, neurite outgrowth and cell survival assays

Primary dissociated DRG neuronal cultures were prepared from adult C57Bl/6 mice^15,33,79,80^. Briefly, DRGs were dissected, and incubated with collagenase dispase II (Roche Diagnostics), and dissociated. Two thousand DRG neurons were plated onto poly-d-lysine and laminin (Sigma-Aldrich)-coated 8-well chamber slide (Millipore), and grown in full NB medium. Small molecules at various doses were added to the cultures after 1 h of plating, and grown for 17 h. PFA-fixed neurons that bear neurite were identified by β-tubulin III immunostaining, and 30 non-overlapping images were taken at 10× magnifications using an epifluorescence microscope (Nikon Eclipse 90i) equipped with a motorized stage. Total neurite length was measured from at least 250 DRG neurons per treatment condition by a fully automated software program NeuroMath (Weizmann Institute of Science). Data were obtained from 3 independent experiments repeated in duplicates.

Cell survival assay was performed using WST-1 reagents according to the manufacturer’s instructions (ClonTech). Briefly, DRG neurons were plated at a density of 500 neurons per well in 96-well plates coated with poly-D-lysine (100 µg/ml) and laminin (10 µg/ml) (Sigma-Aldrich) containing full NB medium. After treatments, 10 µl of WST-1 reagent was added to each well and incubated for 3.5 h. The absorbance at 460 nm was determined using a microplate reader (BioTek Powerwave XS MQX200R). Data were obtained from three independent experiments repeated in triplicate.

### Sciatic nerve pinch test and quantification of in vivo axon regeneration

Sciatic nerve pinch test was used to quantify the rate of in vivo axon regeneration. Left sciatic nerves were crushed with smooth forceps for 15 s^15,16^. Glycopyrrolate (2 mg/kg) or mexiletine (10 mg/kg) was dissolved in 0.9% saline, and intraperitoneally injected immediately after SNC injury for 3 consecutive days. On day 3 post-injury, mice under 1% isoflurane were tested by starting distally; a series of pinches was delivered to sciatic nerve moving proximally toward injury site. Rate of in vivo axon regeneration was determined by measuring distance from injury site to the most distal point on the nerve that produces a reflex withdrawal when pinched. The observers were blinded to the treatments. Pinch test results were validated by mean number of GAP-43-positive fibers determined from 6–9 longitudinal 12-μm-thick cryosections per mouse^15,16^. PFA-fixed sciatic nerves were cryosectioned, and immunostained with anti-GAP-43 antibody (Millipore) and Alexa Fluor 488-conjugated secondary antibody (Molecular Probes) for quantifying regenerating axons.

### Optic nerve crush (ONC) injury

ONC injury was performed on ketamine (100 mg/kg)/xylazine (10 mg/kg) anesthetized adult male mice^9–12,37,44,81^. The left optic nerve was exposed intraorbitally and crushed with jeweler’s forceps for 5 s at 1 mm from the optic disc. Glycopyrrolate or mexiletine at a dose of 1 µg/µl in 0.9% saline (1 µl) was intravitreally injected at a slow rate of 0.2 µl/min (Pump11, Harvard Apparatus) immediately after ONC. Intravitreal injections were performed once weekly for 2 weeks on days 0 and 7 and 4 weeks on days 0, 7, 14, and 21. Mice were also injected daily intraperitoneally with glycopyrrolate (2 mg/kg) and/or mexiletine (10 mg/kg) for 7 consecutive days after ONC. On day 12 post-injury, mice were injected intravitreally with cholera toxin subunit B (CTB)/Alexa Fluor to trace regenerating axons (see Fig. 7a for a schematic experimental paradigm).

### RNA extraction and quantitative real-time polymerase chain reaction (qPCR) analysis

Total RNA was extracted from L4/5 DRGs or whole retinae after LBP, glycopyrrolate or mexiletine treatment using Trizol reagent (Invitrogen)^39,82^. After the determination of RNA concentration, total RNA was reverse transcribed using PrimeScript RT Master Mix (Takara). Triplicate qPCR reactions were performed using TB Green Master Mix (Takara) on a QuantStudio 12 K Flex Real-Time PCR system. The Ct-values were recorded and the relative fold-change of each gene was calculated using 2^−ΔΔCt^ method^39,82^. *Gapdh* was used for normalization. All the primers used in this study was listed in Supplementary Table 2.

### Retinal ganglion cells (RGCs) survival assay

On days 14 and 28 following ONC injury, the mice were transcardially perfused with 0.9% saline followed by 4% PFA, frozen in OCT compounds and cut into 20µm-thick serial transverse retinal cryosections^5,12,44^. The cryosections were blocked and immunostained with anti-RBPMS antibodies (Abcam) and secondary antibodies conjugated with Alexa Fluor 647 (Molecular Probes). Images were taken at 40× magnifications using Carl Zeiss LSM 880 confocal microscope equipped with AiryScan Fast mode and a motorized stage. RBPMS-positive RGCs were counted in every fifth section per contralateral and ipsilateral retinae (i.e. 3–5 sections per retina) using the cell counter plugin from ImageJ software. Changes in RGC density were calculated as percentage of RGC survival in the ipsilateral retinae, normalized to the uninjured contralateral retinae from the same animal^83^. RGC counting was done blinded to surgery and treatments.

To examine whether intravitreal injections of LBP-induced macrophage infiltration, 20 µm-thick serial cryosections of retinae were blocked, incubated with anti-CD68 (Abcam) antibodies, anti-βIII-tubulin primary antibodies (Abcam), and secondary antibodies conjugated with Alexa Fluor 488 or 555 (Molecular Probes) accordingly. Images were taken at 40× magnification using Nikon AXR confocal microscope equipped with a motorized stage and a Galvano scanner.

### Anterograde labeling and quantification of regenerating axons

To quantify regenerating axons by anterograde labeling, 2 µg of recombinant CTB conjugated with Alexa Fluor 555 was injected intravitreally two days before transcardial perfusion with 4% PFA. For the spared axon studies, 2 µg of recombinant CTB conjugated with Alexa Fluor 488 was injected intravitreally two days before the ONC, and CTB-555 were intravitreally injected to trace regenerating RGC axons one day after the ONC^47^ and glycopyrrolate treatment. Whole optic nerves were dissected out, post-fixed with 4% PFA, and cleared^4^. Briefly, optic nerves were incubated with increasing concentrations of ethanol (i.e. 50%, 80 and 95%) for 20 min at room temperature. The nerves were then dehydrated for overnight with 100% ethanol, followed by 100% hexane for 3 h at room temperature, and finally cleared in a 1:2 mixture of benzyl alcohol and benzyl benzoate (BABB) allowing the remaining alcohol to evaporate. The cleared nerves were mounted on a microscope slide using the BABB solution^4^, and imaged at 20× magnifications using Carl Zeiss LSM 880 confocal microscope equipped with AiryScan Fast mode and a motorized stage, with optical sections at 1.7 µm. The images were stitched and maximum projected using ZEN2.3 Blue software (Carl Zeiss).

To quantify regenerating axons, mean number of CTB-positive axons were estimated by counting the number of CTB-positive axons that extended past the crush injury site in 3–5 optical sections (10µm-thick) per mouse. The cross-sectional width of optic nerve (i.e. diameter of the nerve) was measured at the point where the counting was performed. The total number of regenerating axons (Σa_*d*_) extending to the distance *d* was determined using the following equation^9^: Σa_*d*_ = πr^2^ x [average axons/mm]/*t*; *t* = thickness of section (i.e. 10 µm). The average number of regenerating axons at each nerve segment was determined from at least 5–7 mice per treatment group. The representative images of whole optic nerve were stitched and maximum intensity projection using ZEN Blue software (Carl Zeiss) was also performed.

To visualize regenerating axons at multiple subcortical visual targets, whole brains were harvested 2 days after intravitreal injection of CTB-555 at week 6 following ONC, and then post-fixed with 4% PFA, cut into 40 µm thick coronal sections, and counterstained with DAPI for image analysis. The SCN was identified by a cluster of DAPI-stained nuclei above the optic chiasm and adjacent to the third ventricle. The Allen Mouse Brain Atlas was used for the identification of OT, vLGN, dLGN, OPN, and SC. Images were taken at 40× magnification using Nikon AXR confocal microscope equipped with a motorized stage and a Galvano scanner, and maximally projected using NIS-Elements software (Nikon).

To measure the fluorescent intensity of CTB-positive regenerating axons at multiple subcortical visual targets, the area of SCN, OT, vLGN, dLGN, OPN, and SC was manually outlined and defined as the region of interest (ROI). The integral fluorescent intensity of CTB-positive regenerating RGC axons in every fifth section was measured using ImageJ software (NIH) and normalized with the area of ROI^74^. The average fluorescent intensity of regenerating axons was determined from 3 mice per treatment group.

### Optogenetic stimulation of RGCs and eye-evoked LFP recording in the superior colliculus (SC)

Two weeks before the eye-evoked LFP recording, adeno-associated virus (AAV) encoding channel rhodopsin (ChR2-mCherry) were intravitreally injected into the left eye at a viral titer of 5 × 10^12^ vg/ml (see Fig. 9a for detailed experimental paradigm). For precise stereotaxic electrode placement, a midline incision (10 mm) was made to locate the bregma and lambda sutures. The SC was exposed and visualized by craniotomy, which was 5-mm lateral and rostral to the lambda suture of the right hemisphere. Customized multi-electrode arrays (MEAs) 16-channel electrodes were fabricated with nichrome wire (Gamry instruments) and tested for impedance before use. MEA electrode was placed unilaterally into the SC in the right hemisphere at the following co-ordinates relative to the bregma: anteroposterior (AP), −4.25 mm; mediolateral (ML), −1.0 mm; and dorsoventral (DV), −1.0 mm from the skull surface. The grounding wire was secured to the frontal bone with screws, and the reference wire was inserted between the dura and skull.

Optogenetic activation of RGCs were performed for eye-evoked LFP recording in the SC. The injured (left) eye was first cleaned, and eye gel was applied to prevent the corneas from drying during the optical stimulation. To enhance nerve conduction of regenerating axons, mice were injected intraperitoneally with 4 mg/kg of an FDA-approved voltage-gated potassium channel blocker (4-aminopyridine) 3 h before the recording^49^. Light stimuli were delivered via an optic fiber (blue laser at 473 nm) and illuminated at the periphery of the left injured eye with 10 ms pulse and constant light power at 1 mW. LFP was recorded from the SC on the right hemisphere using Blackrock microsystem with pre-set analog filters (high-pass filter: 0.3 Hz, low-pass filter: 100 Hz); sampled at 100 Hz; and digitally filtered (low-pass FIR filter: 25 Hz). No LFP signal was detected when the laser was off or not directly on the surface of the eye. LFP was recorded from the SC on the right hemisphere of uninjured mice as a positive control. Data analysis was performed using Spike2 and customized MATLAB programs. The maximal amplitudes of each mouse were calculated from at least 200 peaks in synchrony with the laser stimulus onset to obtain the LFP signal. Data was obtained from at least 4–6 mice per treatment group in 3 separate experiments.

### Pupillary light reflex (PLR)

To assess restoration of visual function after ONC, we performed PLR^50,64^. Briefly, the mice were first dark-adapted for at least 1 h to allow maximal pupil dilation before the test. During the entire course of the experiment, unanesthetized dark-adapted mice were hand-restrained, and a digital camera (Logitech) was used to record from the injured eye for 30 s under a 470-nm LED light source (30 lux). The percentage of pupil constriction was calculated as the percentage change in pupil size at 30 s after the initiation of the light stimulus relative to the fully dilated pupil size before the light stimulation. The video was first converted into still images using VLC Player, and the diameter of fully dilated pupil after dark adaptation and maximal pupil constriction was measured by ImageJ software (NIH). Data was obtained from 4–6 mice per group.

### Reporting summary

Further information on research design is available in the Nature Research Reporting Summary linked to this article.

## Supplementary information

Supplementary Information

REPORTING SUMMARY

## Data Availability

All the data are available in the main text or supplementary materials. The materials are available from the corresponding author upon reasonable request. The microarray data reported in the current study was deposited in Gene Expression Omnibus (GEO) under the accession number GSE200112.

## Code availability

The MATLAB code that used in current study is available from the corresponding author upon reasonable request.

## References

[1] Schwab ME, Strittmatter SM (2014). Nogo limits neural plasticity and recovery from injury. Curr. Opin. Neurobiol..

[2] Mahar M, Cavalli V (2018). Intrinsic mechanisms of neuronal axon regeneration. Nat. Rev. Neurosci..

[3] So KF, Aguayo AJ (1985). Lengthy regrowth of cut axons from ganglion cells after peripheral nerve transplantation into the retina of adult rats. Brain Res..

[4] Cartoni R (2016). The mammalian-specific protein Armcx1 regulates mitochondrial transport during axon regeneration. Neuron.

[5] Norsworthy MW (2017). Sox11 expression promotes regeneration of some retinal ganglion cell types but kills others. Neuron.

[6] Sun F (2011). Sustained axon regeneration induced by co-deletion of PTEN and SOCS3. Nature.

[7] Belin S (2015). Injury-induced decline of intrinsic regenerative ability revealed by quantitative proteomics. Neuron.

[8] Nawabi H (2015). Doublecortin-like kinases promote neuronal survival and induce growth cone reformation via distinct mechanisms. Neuron.

[9] Park KK (2008). Promoting axon regeneration in the adult CNS by modulation of the PTEN/mTOR pathway. Science.

[10] Hu Y (2012). Differential effects of unfolded protein response pathways on axon injury-induced death of retinal ganglion cells. Neuron.

[11] Park KK, Liu K, Hu Y, Kanter JL, He Z (2010). PTEN/mTOR and axon regeneration. Exp. Neurol..

[12] Yang L (2014). The mTORC1 effectors S6K1 and 4E-BP play different roles in CNS axon regeneration. Nat. Commun..

[13] Wang J (2013). Pleiotropic molecules in axon regeneration and neuroinflammation. Exp.Neurol.

[14] Kim JE, Liu BP, Park JH, Strittmatter SM (2004). Nogo-66 receptor prevents raphespinal and rubrospinal axon regeneration and limits functional recovery from spinal cord injury. Neuron.

[15] Ma CH (2011). Accelerating axonal growth promotes motor recovery after peripheral nerve injury in mice. J. Clin. Invest..

[16] Ma CH (2011). The BMP coreceptor RGMb promotes while the endogenous BMP antagonist noggin reduces neurite outgrowth and peripheral nerve regeneration by modulating BMP signaling. J. Neurosci..

[17] Ma CH, Taylor JS (2010). Trophic responsiveness of purified postnatal and adult rat retinal ganglion cells. Cell Tissue Res..

[18] Lang BT (2014). Pleiotropic molecules in axon regeneration and neuroinflammation. Exp. Neurol..

[19] Bampton ET, Ma CH, Tolkovsky AM, Taylor JS (2005). Osteonectin is a Schwann cell-secreted factor that promotes retinal ganglion cell survival and process outgrowth. Eur. J. Neurosci..

[20] Ma CH, Bampton ET, Evans MJ, Taylor JS (2010). Synergistic effects of osteonectin and brain-derived neurotrophic factor on axotomized retinal ganglion cells neurite outgrowth via the mitogen-activated protein kinase-extracellular signal-regulated kinase 1/2 pathways. Neuroscience.

[21] Ma CH, Palmer A, Taylor JS (2009). Synergistic effects of osteonectin and NGF in promoting survival and neurite outgrowth of superior cervical ganglion neurons. Brain Res..

[22] Asthana P, Zhang G, Sheikh KA, Him Eddie Ma C (2021). Heat shock protein is a key therapeutic target for nerve repair in autoimmune peripheral neuropathy and severe peripheral nerve injury. Brain Behav. Immun..

[23] Li C, Samulski RJ (2020). Engineering adeno-associated virus vectors for gene therapy. Nat. Rev. Genet..

[24] Shi Z (2017). Neuroprotective mechanisms of lycium barbarum polysaccharides against ischemic insults by regulating NR2B and NR2A containing NMDA receptor signaling pathways. Front Cell Neurosci..

[25] Chu PH, Li HY, Chin MP, So KF, Chan HH (2013). Effect of lycium barbarum (wolfberry) polysaccharides on preserving retinal function after partial optic nerve transection. PLoS ONE.

[26] Chan HC (2007). Neuroprotective effects of Lycium barbarum Lynn on protecting retinal ganglion cells in an ocular hypertension model of glaucoma. Exp. Neurol..

[27] Mi XS (2012). Protection of retinal ganglion cells and retinal vasculature by Lycium barbarum polysaccharides in a mouse model of acute ocular hypertension. PloS ONE.

[28] Yang D (2012). Lycium barbarum extracts protect the brain from blood-brain barrier disruption and cerebral edema in experimental stroke. PloS ONE.

[29] Yu MS (2007). Characterization of the effects of anti-aging medicine Fructus lycii on beta-amyloid peptide neurotoxicity. Int. J. Mol. Med..

[30] Yu MS (2005). Neuroprotective effects of anti-aging oriental medicine Lycium barbarum against beta-amyloid peptide neurotoxicity. Exp. Gerontol..

[31] Ho YS (2009). Polysaccharides from wolfberry antagonizes glutamate excitotoxicity in rat cortical neurons. Cell. Mol. Neurobiol..

[32] Ho YS (2010). Neuroprotective effects of polysaccharides from wolfberry, the fruits of Lycium barbarum, against homocysteine-induced toxicity in rat cortical neurons. J. Alzheimer’s Dis..

[33] Au NP (2016). Ciguatoxin reduces regenerative capacity of axotomized peripheral neurons and delays functional recovery in pre-exposed mice after peripheral nerve injury. Sci. Rep..

[34] Asthana P (2018). Pacific ciguatoxin induces excitotoxicity and neurodegeneration in the motor cortex via Caspase 3 activation: implication for irreversible motor deficit. Mol. Neurobiol..

[35] Peng S, Shi Z, Su H, So KF, Cui Q (2016). Increased production of omega-3 fatty acids protects retinal ganglion cells after optic nerve injury in mice. Exp. Eye Res..

[36] Lamb J (2006). The connectivity map: using gene-expression signatures to connect small molecules, genes, and disease. Science.

[37] Chandran V (2016). A systems-level analysis of the peripheral nerve intrinsic axonal growth program. Neuron.

[38] Yang PM, Chou CJ, Tseng SH, Hung CF (2017). Bioinformatics and in vitro experimental analyses identify the selective therapeutic potential of interferon gamma and apigenin against cervical squamous cell carcinoma and adenocarcinoma. Oncotarget.

[39] Tam WY, Ma CHE (2014). Bipolar/rod-shaped microglia are proliferating microglia with distinct M1/M2 phenotypes. Sci. Rep..

[40] Ledeboer A (2007). Intrathecal interleukin-10 gene therapy attenuates paclitaxel-induced mechanical allodynia and proinflammatory cytokine expression in dorsal root ganglia in rats. Brain Behav. Immun..

[41] Niemi JP, DeFrancesco-Lisowitz A, Cregg JM, Howarth M, Zigmond RE (2016). Overexpression of the monocyte chemokine CCL2 in dorsal root ganglion neurons causes a conditioning-like increase in neurite outgrowth and does so via a STAT3 dependent mechanism. Exp. Neurol..

[42] Kumar, G. et al. Acute exposure to pacific ciguatoxin reduces electroencephalogram activity and disrupts neurotransmitter metabolic pathways in motor cortex. *Mol. Neurobiol.*10.1007/s12035-016-0093-y (2016). 10.1007/s12035-016-0093-y 27613284

[43] Smith PD (2009). SOCS3 deletion promotes optic nerve regeneration in vivo. Neuron.

[44] Miao L (2016). mTORC1 is necessary but mTORC2 and GSK3beta are inhibitory for AKT3-induced axon regeneration in the central nervous system. Elife.

[45] Dhande OS, Stafford BK, Lim JA, Huberman AD (2015). Contributions of retinal ganglion cells to subcortical visual processing and behaviors. Annu. Rev. Vis. Sci..

[46] de Lima S (2012). Full-length axon regeneration in the adult mouse optic nerve and partial recovery of simple visual behaviors. Proc. Natl Acad. Sci. USA.

[47] Lim JH (2016). Neural activity promotes long-distance, target-specific regeneration of adult retinal axons. Nat. Neurosci..

[48] Fischer D, Harvey AR, Pernet V, Lemmon VP, Park KK (2017). Optic nerve regeneration in mammals: regenerated or spared axons. Exp. Neurol..

[49] Bei F (2016). Restoration of visual function by enhancing conduction in regenerated axons. Cell.

[50] Sweeney NT, Tierney H, Feldheim DA (2014). Tbr2 is required to generate a neural circuit mediating the pupillary light reflex. J. Neurosci..

[51] Collaborators GBDN (2019). Global, regional, and national burden of neurological disorders, 1990-2016: a systematic analysis for the Global Burden of Disease Study 2016. Lancet Neurol..

[52] Sarkies N (2004). Traumatic optic neuropathy. Eye.

[53] Steinsapir KD, Goldberg RA (2011). Traumatic optic neuropathy: an evolving understanding. Am. J. Ophthalmol..

[54] Levin LA (1999). The treatment of traumatic optic neuropathy: the International Optic Nerve Trauma Study. Ophthalmology.

[55] Jarvis B, Coukell AJ (1998). Mexiletine. Drugs.

[56] Wallace MS, Magnuson S, Ridgeway B (2000). Efficacy of oral mexiletine for neuropathic pain with allodynia: a double-blind, placebo-controlled, crossover study. Reg. Anesth. Pain. Med..

[57] Subramanian N (2012). Role of Na(v)1.9 in activity-dependent axon growth in motoneurons. Hum. Mol. Genet..

[58] Chabicovsky M (2019). Pharmacology, toxicology and clinical safety of glycopyrrolate. Toxicol. Appl Pharm..

[59] Mirakhur RK, Dundee JW (1983). Glycopyrrolate: pharmacology and clinical use. Anaesthesia.

[60] Calcutt NA (2017). Selective antagonism of muscarinic receptors is neuroprotective in peripheral neuropathy. J. Clin. Invest..

[61] Li Y (2017). Mobile zinc increases rapidly in the retina after optic nerve injury and regulates ganglion cell survival and optic nerve regeneration. Proc. Natl Acad. Sci. USA.

[62] Zuchner T, Schliebe N, Schliebs R (2006). Zinc uptake is mediated by M1 muscarinic acetylcholine receptors in differentiated SK-SH-SY5Y cells. Int. J. Dev. Neurosci..

[63] Strang CE, Renna JM, Amthor FR, Keyser KT (2010). Muscarinic acetylcholine receptor localization and activation effects on ganglion response properties. Invest. Ophthalmol. Vis. Sci..

[64] Chen SK, Badea TC, Hattar S (2011). Photoentrainment and pupillary light reflex are mediated by distinct populations of ipRGCs. Nature.

[65] Ecker JL (2010). Melanopsin-expressing retinal ganglion-cell photoreceptors: cellular diversity and role in pattern vision. Neuron.

[66] Guler AD (2008). Melanopsin cells are the principal conduits for rod-cone input to non-image-forming vision. Nature.

[67] Rheaume BA (2018). Single cell transcriptome profiling of retinal ganglion cells identifies cellular subtypes. Nat. Commun..

[68] Tran NM (2019). Single-cell profiles of retinal ganglion cells differing in resilience to injury reveal neuroprotective genes. Neuron.

[69] Duan X (2015). Subtype-specific regeneration of retinal ganglion cells following axotomy: effects of osteopontin and mTOR signaling. Neuron.

[70] Bray ER (2019). Thrombospondin-1 mediates axon regeneration in retinal ganglion cells. Neuron.

[71] Perez de Sevilla Muller L, Sargoy A, Rodriguez AR, Brecha NC (2014). Melanopsin ganglion cells are the most resistant retinal ganglion cell type to axonal injury in the rat retina. PLoS ONE.

[72] Robinson GA, Madison RD (2004). Axotomized mouse retinal ganglion cells containing melanopsin show enhanced survival, but not enhanced axon regrowth into a peripheral nerve graft. Vis. Res..

[73] Li S (2016). Promoting axon regeneration in the adult CNS by modulation of the melanopsin/GPCR signaling. Proc. Natl Acad. Sci. USA.

[74] Li S (2015). Injured adult retinal axons with Pten and Socs3 co-deletion reform active synapses with suprachiasmatic neurons. Neurobiol. Dis..

[75] Luo X (2013). Three-dimensional evaluation of retinal ganglion cell axon regeneration and pathfinding in whole mouse tissue after injury. Exp. Neurol..

[76] Wang Z (2020). Intravitreal injection of human retinal progenitor cells for treatment of retinal degeneration. Med Sci. Monit..

[77] Satarian L (2017). Intravitreal injection of bone marrow mesenchymal stem cells in patients with advanced retinitis pigmentosa; a safety study. J. Ophthalmic Vis. Res..

[78] Do DV, Rhoades W, Nguyen QD (2020). Pharmacokinetic study of intravitreal aflibercept in humans with neovascular age-related macular degeneration. Retina.

[79] Au NP, Fang Y, Xi N, Lai KW, Ma CH (2014). Probing for chemotherapy-induced peripheral neuropathy in live dorsal root ganglion neurons with atomic force microscopy. Nanomedicine.

[80] Chine VB, Au NPB, Ma CHE (2019). Therapeutic benefits of maintaining mitochondrial integrity and calcium homeostasis by forced expression of Hsp27 in chemotherapy-induced peripheral neuropathy. Neurobiol. Dis..

[81] Li L (2020). Longitudinal morphological and functional assessment of RGC neurodegeneration after optic nerve crush in mouse. Front Cell Neurosci..

[82] Tam WY, Au NP, Ma CH (2016). The association between laminin and microglial morphology in vitro. Sci. Rep..

[83] Zhang Y (2019). Elevating growth factor responsiveness and axon regeneration by modulating presynaptic inputs. Neuron.

