# Supplementary material for: Clinically relevant small-molecule promotes nerve repair and visual function recovery
Source: NPJ Regen Med. 2022 Oct 1;7:50. doi: 10.1038/s41536-022-00233-8 (PMC9526721; doi:10.1038/s41536-022-00233-8)
Supplement: Supplementary file 1 — Supplementary Information [file 41536_2022_233_MOESM1_ESM.pdf]

Supplementary Information for

Clinically relevant small-molecule promotes nerve repair and visual recovery

Ngan Pan Bennett Au[1]§, Gajendra Kumar[1]§, Pallavi Asthana[1]§, Fuying Gao[2], Riki Kawaguchi[2], Raymond Chuen Chung Chang[3,4], Kwok Fai So[4,5,6], Yang Hu[7], Daniel H. Geschwind[8,9], Giovanni Coppola[8,9], Chi Him Eddie Ma[1,*]

[1]Department of Neuroscience, City University of Hong Kong, Tat Chee Avenue, Hong Kong SAR. [2]Department of Psychiatry, Semel Institute for Neuroscience and Human Behavior, David Geffen School of Medicine, University of California Los Angeles, Los Angeles, CA 90095, USA. [3]Laboratory of Neurodegenerative Diseases, School of Biomedical Sciences, LKS Faculty of Medicine, The University of Hong Kong, Pokfulam, Hong Kong SAR. [4]State Key Laboratory of Brain and Cognitive Sciences, The University of Hong Kong, Pokfulam, Hong Kong SAR. [5]Department of Ophthalmology, The University of Hong Kong, Pokfulam, Hong Kong. [6]Guangdong-Hong Kong-Macau Institute of CNS Regeneration, Jinan University, Guangzhou, China. [7]Department of Ophthalmology, Stanford University School of Medicine, Palo Alto, United States. [8]Program in Neurogenetics, Department of Neurology, David Geffen School of Medicine, University of California, Los Angeles, Los Angeles, CA 90095, USA. [9]Department of Human Genetics, University of California, Los Angeles, Los Angeles, CA 90095, USA.

§ Authors contribute equally

* **Correspondence:** Dr. Chi Him Eddie Ma

[1]Department of Neuroscience, City University of Hong Kong, Tat Chee Avenue, Hong Kong.

Email: eddiema@cityu.edu.hk

Phone: (+852) 3442-9328 Fax: (+852) 3442-0549

Numbers of Supplementary Figures: 13

Numbers of Supplementary Tables: 2

Figure Legends

Supplementary Figure 1. LBP treatment does not alter gene expression of key inflammatory modulators in DRGs. Mice were received oral administrations of LBP (100mg/kg) for 13 consecutive days. Lumbar 4 and 5 (L4/5) DRGs were dissected for qPCR analysis on day 13 after LBP treatment. The gene expression levels of pro-inflammatory cytokines (M1 macrophage marker genes), anti-inflammatory cytokines (M2 macrophage marker genes), and chemokines remained unchanged in DRGs after LBP treatment, suggesting that LBP treatment did not elicit inflammatory responses in the peripheral neurons. Mean ± SEM of triplicate. $P > 0.05$; Student's *t*-test.

Supplementary Figure 2. LBP treatment increases the number of regenerating axons 9 days after a single sciatic nerve crush (SNC). (a) Transverse sections (4 µm-thick) of sciatic nerves were immunostained with anti-neurofilament (NF200) antibody. The total number of axons was quantified at 9 days after SNC and vehicle or 100 mg/kg LBP post-treatment. Scale bar: 50µm. **(b)** Quantification of the total number of regenerating axons in LBP-treated group showed a significant increase in the number of axons located at 10, 20 and 25 mm distal to the injury site in the ipsilateral side, when compared with the PBS-treated (control) group. Mean ± SEM (n = 4-5 per group and 6-8 sections per mouse was quantified using ImageJ software). *$P < 0.05$, **$P < 0.01$, ***$P < 0.001$; Two-way ANOVA followed by *post hoc* Bonferroni's multiple comparison test.

Supplementary Figure 3. LBP treatment increases the number of regenerating axons 13 days after a single sciatic nerve crush (SNC). (a) Transverse sections (4 µm-thick) of sciatic nerves were immunostained with anti-neurofilament (NF200) antibody. The total number of axons was quantified at 9 days after SNC and vehicle or 100 mg/kg LBP post-treatment. Scale bar: 50µm. **(b)** Quantification of the total number of regenerating axons in LBP-treated group

showed a significant increase in the number of axons located from 5 to 25 mm distal to the injury site in the ipsilateral side, when compared with the PBS-treated (control) group. Mean ± SEM (n = 4-5 per group and 6-8 sections per mouse was quantified using ImageJ software). ***$P < 0.001$, Two-way ANOVA followed by *post hoc* Bonferroni's multiple comparison test.

Supplementary Figure 4. LBP treatment increases the number of regenerating axons 17 days after a single sciatic nerve crush (SNC). (a) Transverse sections (4 µm-thick) of sciatic nerves were immunostained with anti-neurofilament (NF200) antibody. The total number of axons was quantified at 9 days after SNC and vehicle or 100 mg/kg LBP post-treatment. Scale bar: 50µm. **(b)** Quantification of the total number of regenerating axons in LBP-treated group showed a significant increase in the number of axons located at 5, 10 and 25 mm distal to the injury site in the ipsilateral side, when compared with the PBS-treated (control) group. Mean ± SEM (n = 4-5 per group and 6-8 sections per mouse was quantified using ImageJ software). *$P < 0.05$, **$P < 0.01$; Two-way ANOVA followed by *post hoc* Bonferroni's multiple comparison test.

Supplementary Figure 5. LBP accelerates the reformation of functional synapses after a single sciatic nerve crush (SNC). (a) Functional neuromuscular junction (NMJ) reinnervation was quantified by counting the overlapping neurofilament (NF200; red) and α-bungarotoxin (BTX; green) immunoreactivity in the distal interosseous muscles of vehicle or 100 mg/kg LBP post-treatment at multiple time-points (days 9, 13 and 17 post-injury). Representative confocal photographs of NMJ reinnervations at days 17 post-injury were shown. Scale bar: 10µm. **(b)** LBP post-treatment (100 mg/kg) increased NMJ reinnervation in the distal interosseous muscles significantly at days 9 and 17 post-injury. Mean ± SEM (n = 3-9 per group); *$P < 0.05$; Student's *t*-test.

Supplementary Figure 6. LBP-treated and vehicle mice fully regenerate axons into distal target muscles after prolonged muscle denervation. (a) Photomicrographs of transverse sections (4 μm-thick) of sciatic nerves through axons located proximal to the injury site and up to 25 mm distal to the injury site 2 months after the last crush in a mouse model of severe peripheral nerve injury. Scale bar 50 μm. **(b)** The total number of NF200-labeled axons was quantified by ImageJ software. There were no significant differences between LBP-treated and vehicle control mice along the sciatic nerves. Mean ± SEM (n = 5 per group and 6-8 sections per mouse was quantified using ImageJ software). Two-way ANOVA followed by *post hoc* Bonferroni's multiple comparison test.

Supplementary Figure 7. LBP enhances target muscle reinnervation two months after severe peripheral nerve injury. (a) Confocal photographs of the distal plantar muscle co-immunostained with anti-α-bungarotoxin (BTX; green) and anti-neurofilament (NF200; red). **(b)** The number of fully innervated NMJs in 100 mg/kg LBP post-treated mice was increased significantly, compared with the vehicle-treated mice. Scale bar 10 μm. Mean ± SEM (n = 4-5 per group); *$P < 0.05$; Student's *t*-test.

Supplementary Figure 8. Oral administration and intravitreal injections of LBP do not trigger neuroinflammation and macrophage infiltration in the retinae. (a) The mice were received oral administrations of LBP (100mg/kg) for 7 consecutive days before receiving intravitreal injections of LBP. At days 0 and 7, 10μg of LBP was intravitreally injected into the left eye without performing any optic nerve crush injuries on the same mice. At the same time, the mice were received oral administrations of LBP (100mg/kg) for 14 consecutive days before tissue harvest on day 14 post-intravitreal injection for qPCR and histological analysis. The gene expression levels of pro-inflammatory cytokines (M1 macrophage marker genes), anti-inflammatory cytokines (M2 macrophage marker genes), and chemokines remained unchanged in retinae after LBP treatment, suggesting that oral administration and intravitreal

injections of LBP did not trigger inflammatory responses. **(b)** The cryosections of retinae were immunostained with anti-CD68 to label resident retinal microglia and infiltrating macrophages. The number of CD68-positive cells remained unchanged after LBP treatment, further confirming that oral administration and intravitreal injections of LBP did not cause microglial activation and macrophage infiltration. Scale bar: 50μm. Mean ± SEM of triplicate. $P > 0.05$; Student's *t*-test.

Supplementary Figure 9. Weighted gene co-expression network analysis (WGCNA) identifies 50 consensus modules and reveals module eigengene correlation after LBP treatment. The graphs indicated the relative expression values of the first principal component, namely eigengene, of each module identified by WGCNA.

Supplementary Figure 10. Module-trait relationship shows correlations between co-expression module eigengenes and LBP treatment. The module name was indicated on the left side of each cell. Each cell was color-coded based on correlation values. Deeper red color indicated a higher positive correlation, and deeper green color indicated a higher negative correlation. The Pearson correlation coefficient was displayed in each cell with corresponding *P*-value highlighted in brackets.

Supplementary Figure 11. Glycopyrrolate and mexiletine recapitulate the LBP gene signature in DRGs. Oral administration of LBP induced down-regulation of 7 genes in DRGs among 9 tested genes tested from microarray analysis. Similar to LBP treatment, glycopyrrolate and mexiletine treatments induced down-regulation of 6 and 5 genes in DRGs, respectively. Mean ± SEM of triplicate. * $P < 0.05$; one-way ANOVA followed by *post hoc* Bonferroni's multiple comparison test. **n.s.**, not significant.

Supplementary Figure 12. Absence of CTB-labeled regenerating RGC axons re-innervation in the major visual targets of vehicle-treated mice at 6 weeks post-ONC. (a-

f) Representative confocal micrographs showed that no CTB-labeled regenerating RGC axons were detected in (a) hypothalamic suprachiasmatic nucleus (SCN), (b) optic tract (OT), (c) thalamic ventral lateral geniculate nucleus (vLGN), (d) dorsal lateral geniculate nucleus (dLGN), (e) olivary pretectal nucleus (OPN), and (f) superior colliculus (SC) of vehicle-treated mice at 6 weeks post-ONC. Scale bars: 50µm.

Supplementary Figure 13. CTB-tracing study demonstrates complete crush of optic nerve with no sprouting of spared RGC axons. (a) Two days before ONC, a recombinant CTB conjugated with Alexa Fluor 488 (CTB-488) was intravitreally injected to label the intact uninjured RGC axons. Immediately after ONC, glycopyrrolate was intravitreally injected into the injured eye, and a recombinant CTB conjugated with Alexa Fluor 555 (CTB-555) was intravitreally injected into the same eye 1 day after ONC to trace the regenerating RGC axons after ONC. Optic nerves were harvested at day 3 post-ONC for image analysis. **(b)** CTB-488-labeled axons were only detected in close vicinity to the crush site (dotted line), but never appeared at the distal nerve beyond the crush site, indicating that the ONC procedures were complete with no sprouting of spared RGC axons. Only CTB-555-labeled regenerating axons (yellow arrowheads) were detected at the distal to the crush site. **(c)** Magnified view of the white inset in (b) showing no overlapping between CTB-488 (intact uninjured axons) and CTB-555 (regenerating axons) fluorescence in the crushed optic nerve at the distal to the crush site. Scale bars: 200µm in (b, c).

Supplementary Table 1. The top 10 most significant differentially expressed genes in mouse DRGs after 13 days oral administration of LBP.

Supplementary Table 2. Summary of primers used in the current study.

Supplementary Figure 1

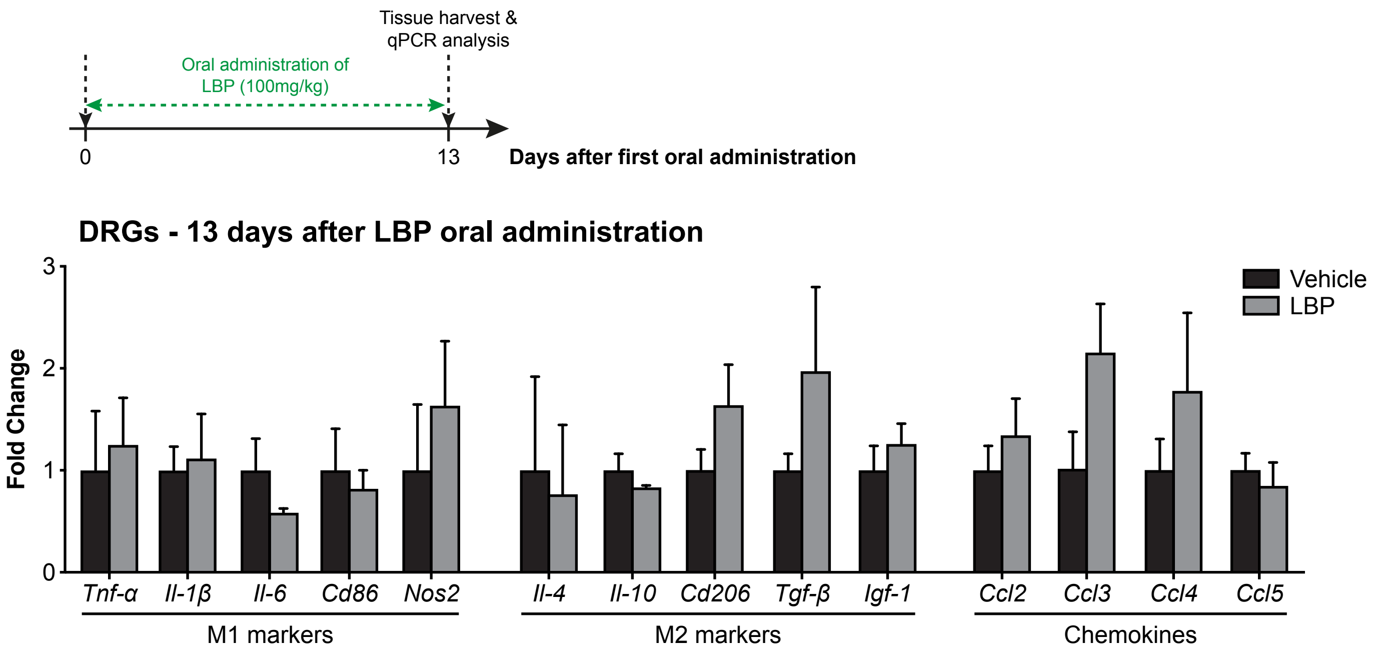

Supplementary Figure 2

a

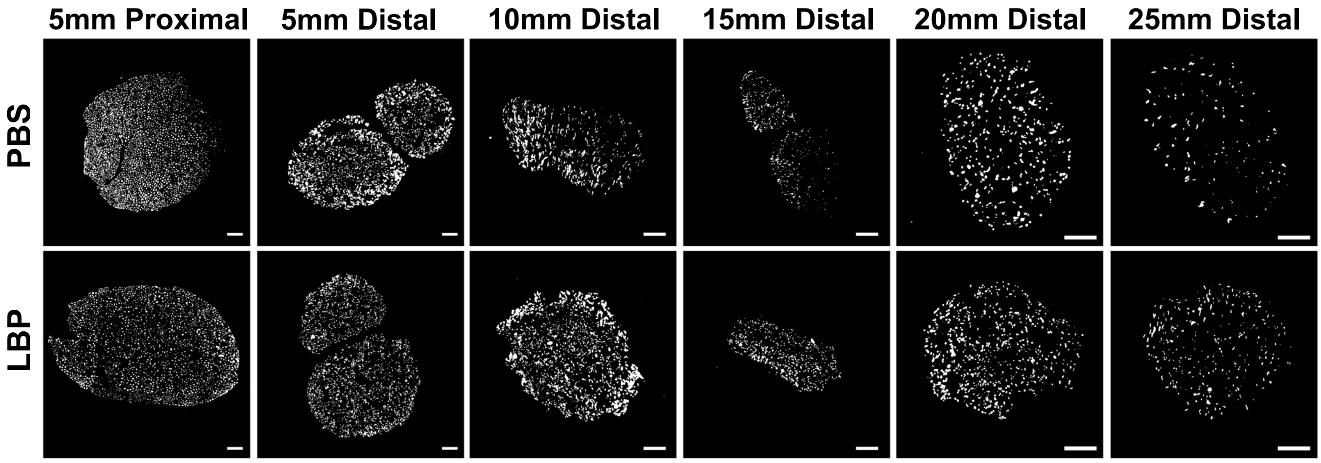

b

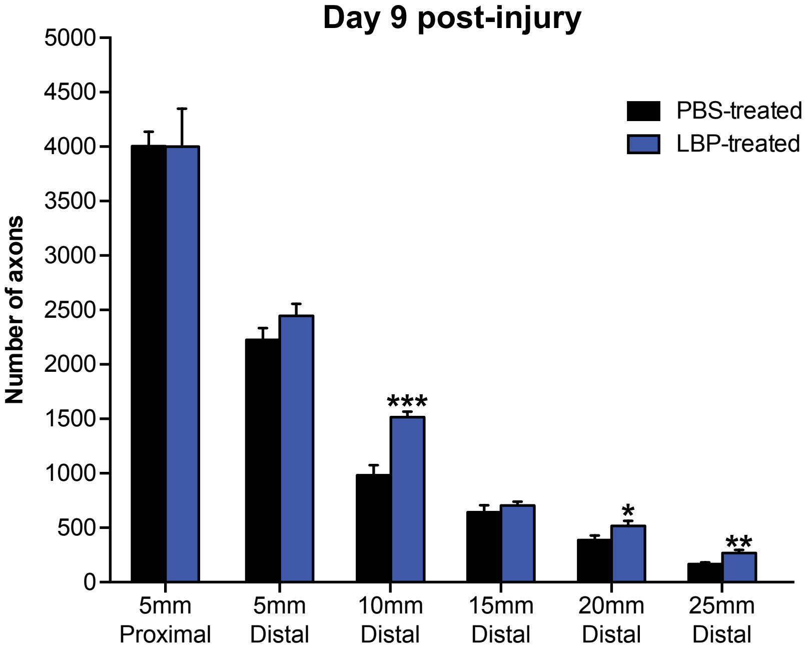

Supplementary Figure 3

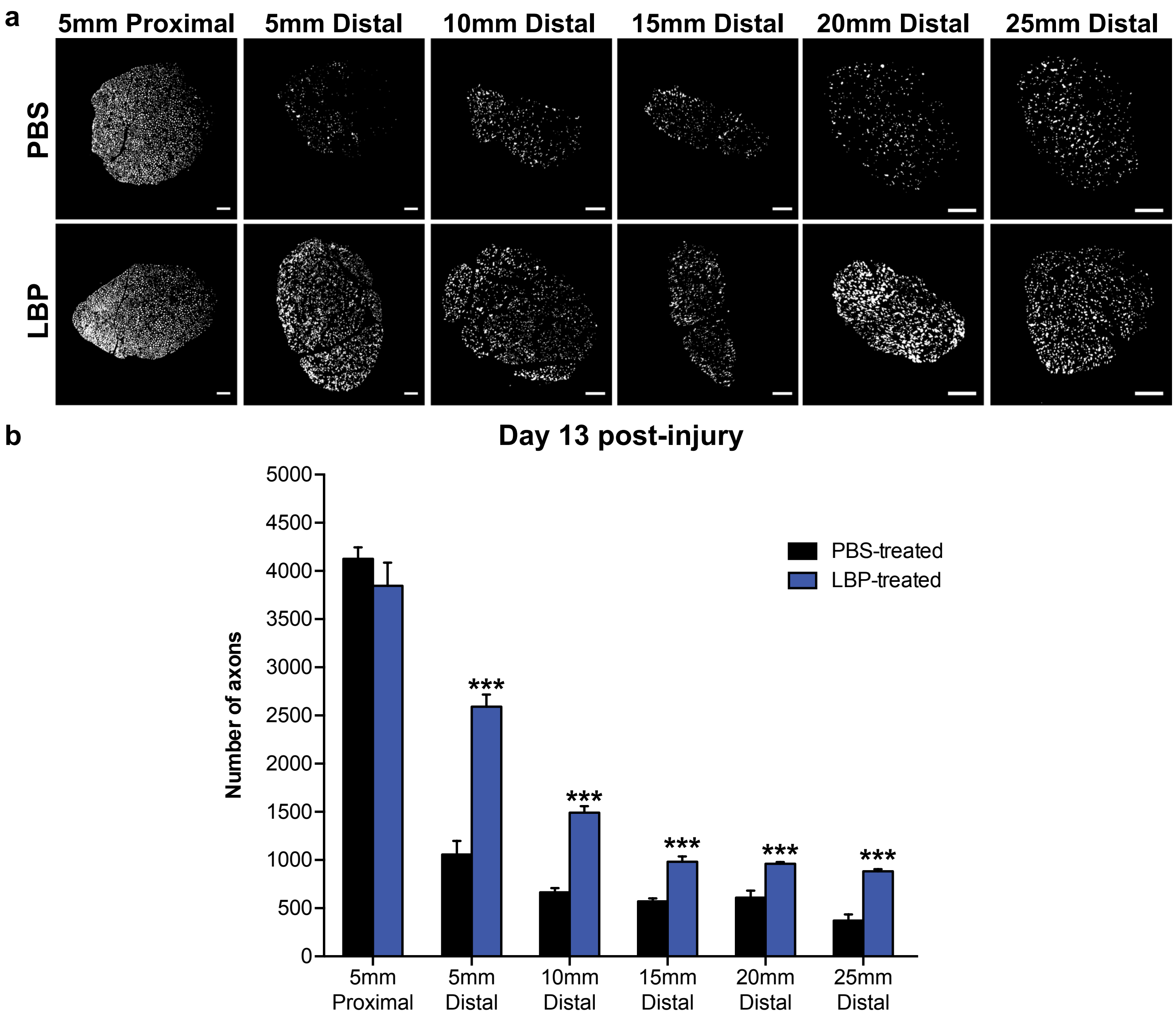

Supplementary Figure 4

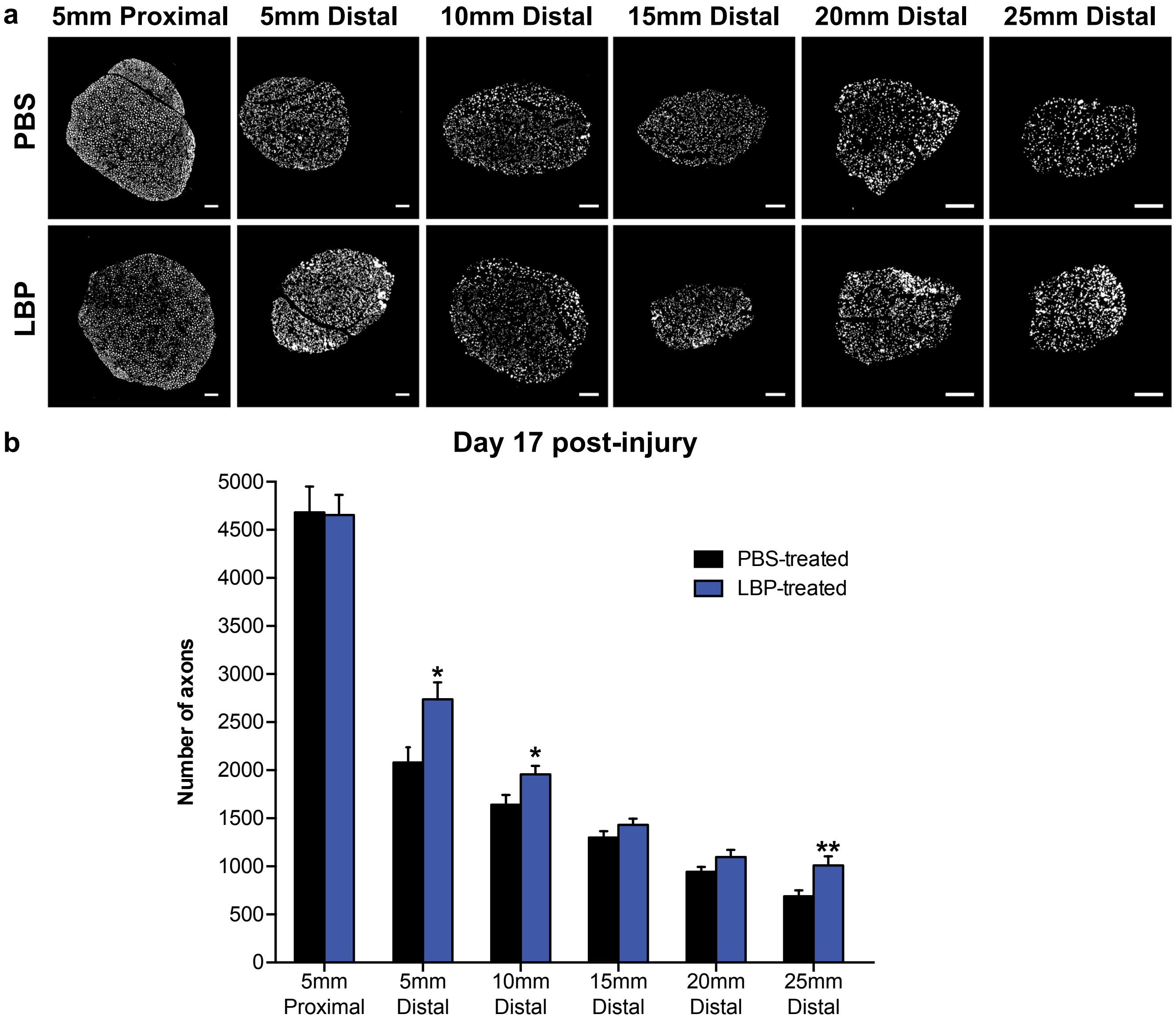

Supplementary Figure 5

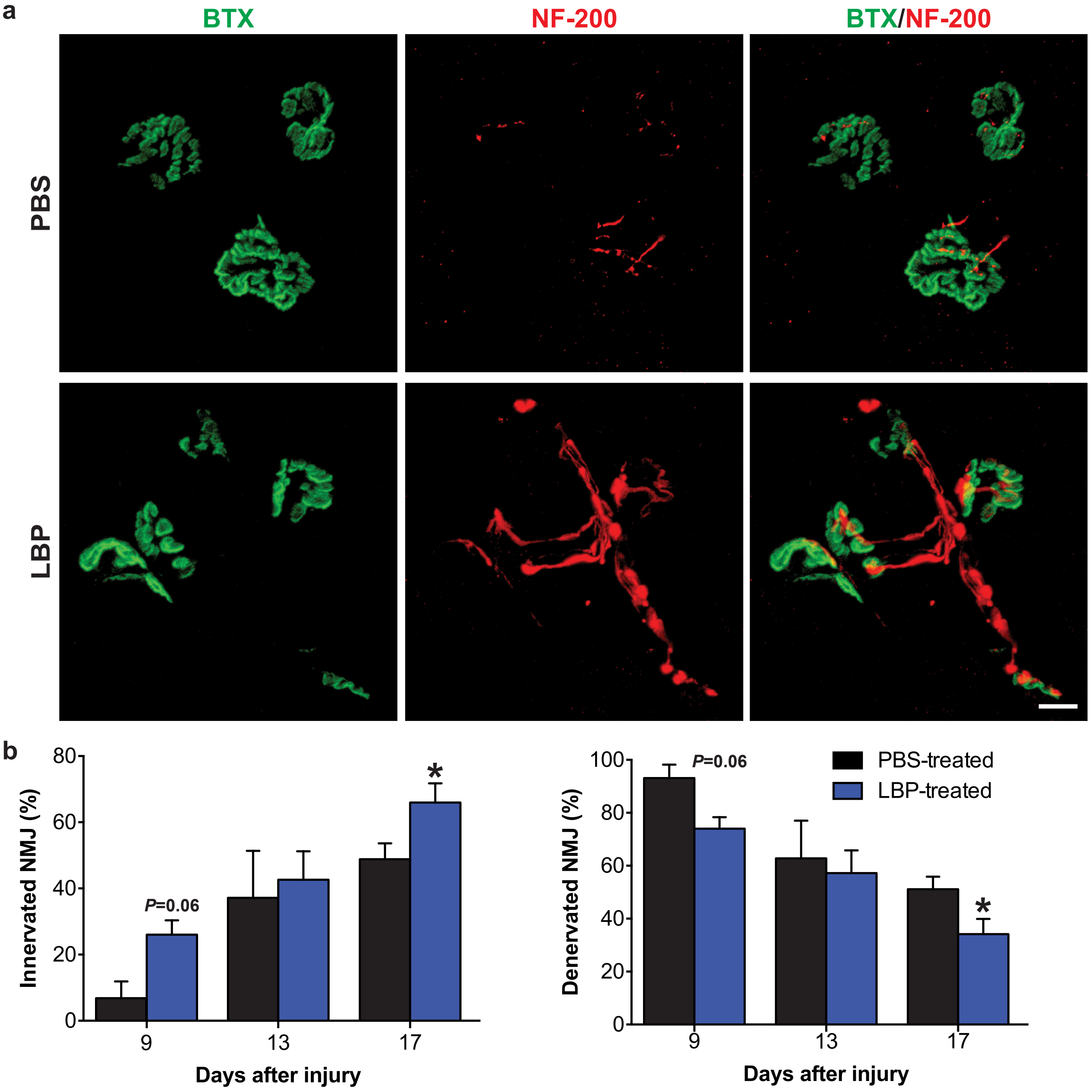

Supplementary Figure 6

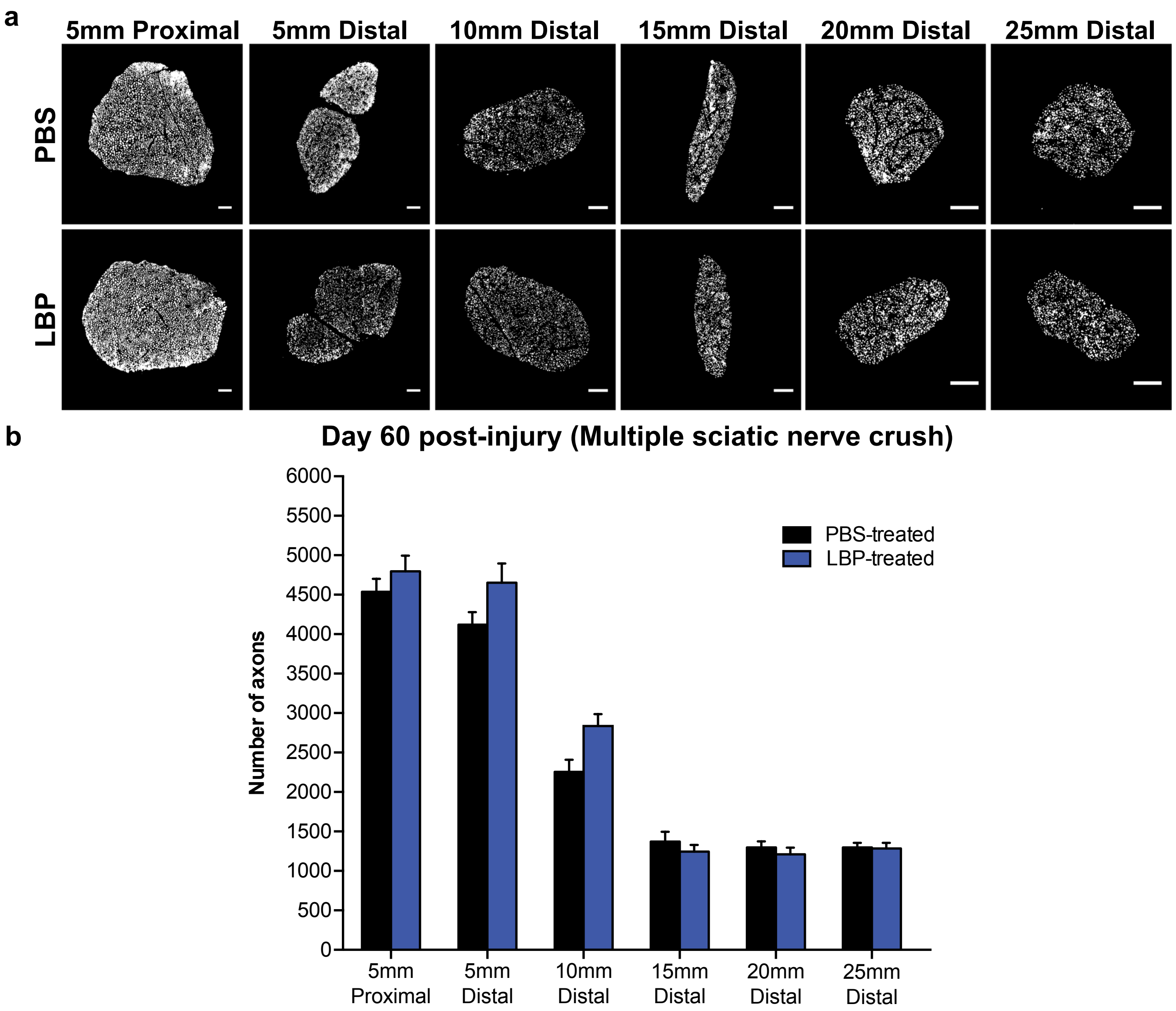

Supplementary Figure 7

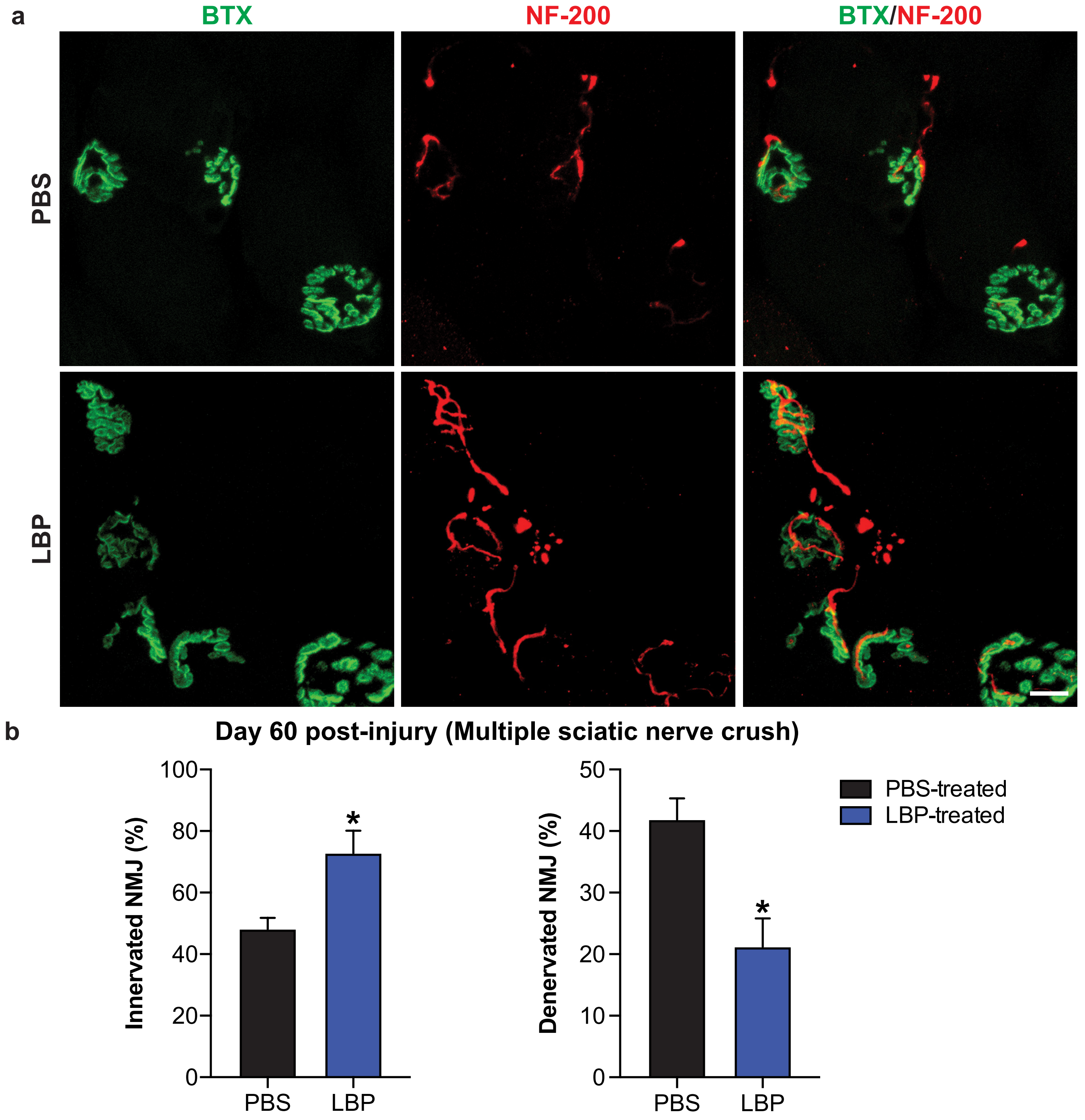

Supplementary Figure 8

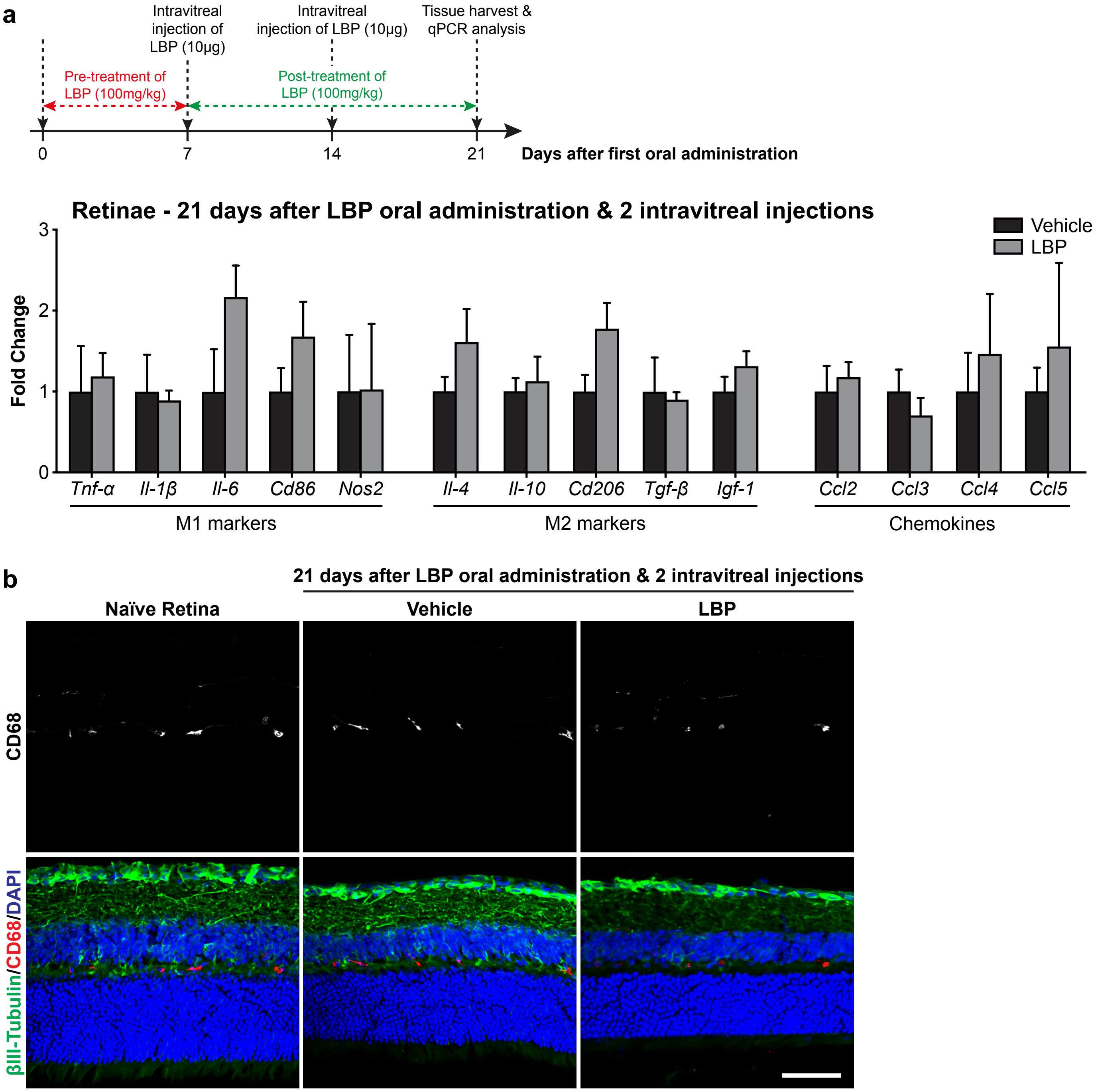

Supplementary Figure 9

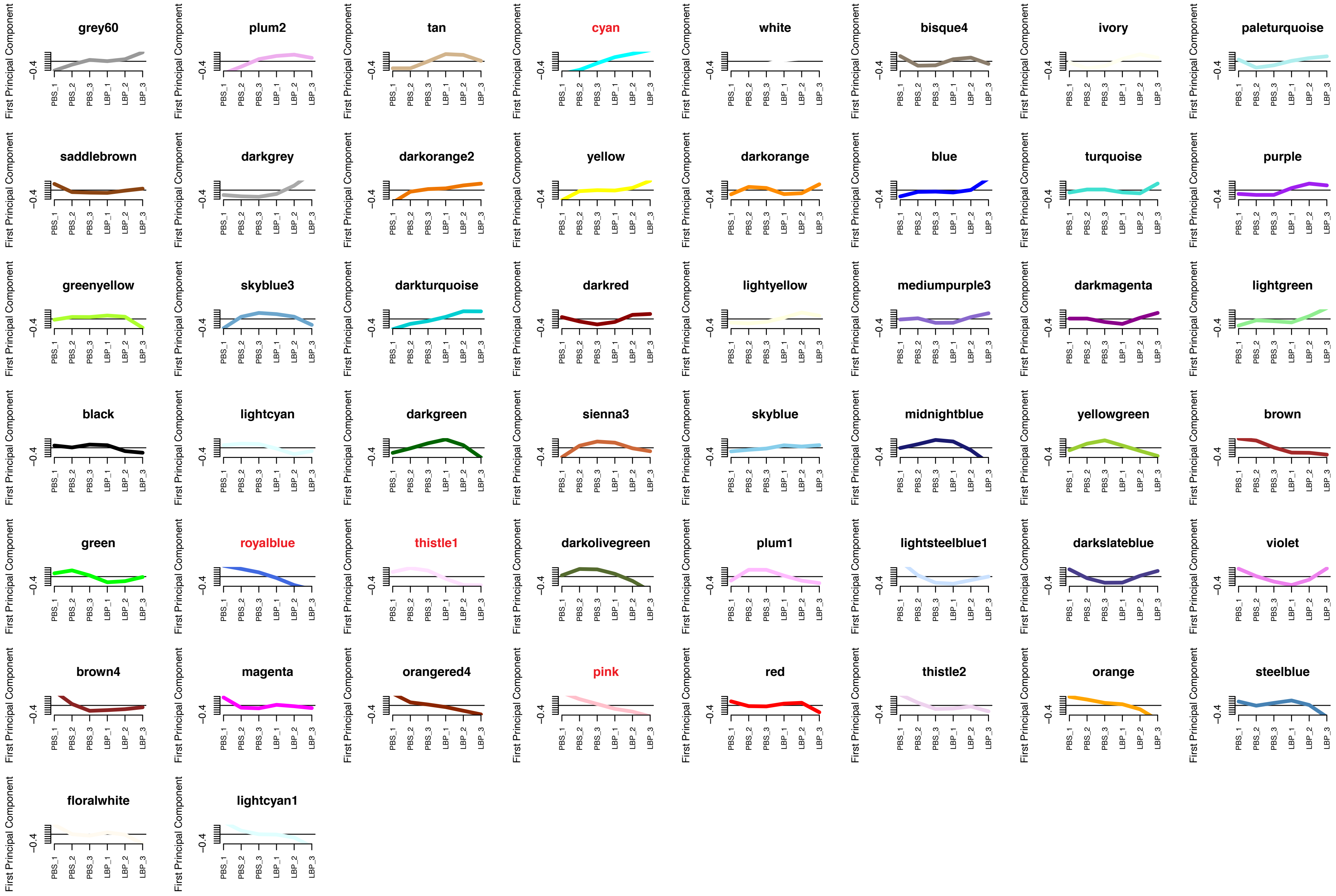

Supplementary Figure 10

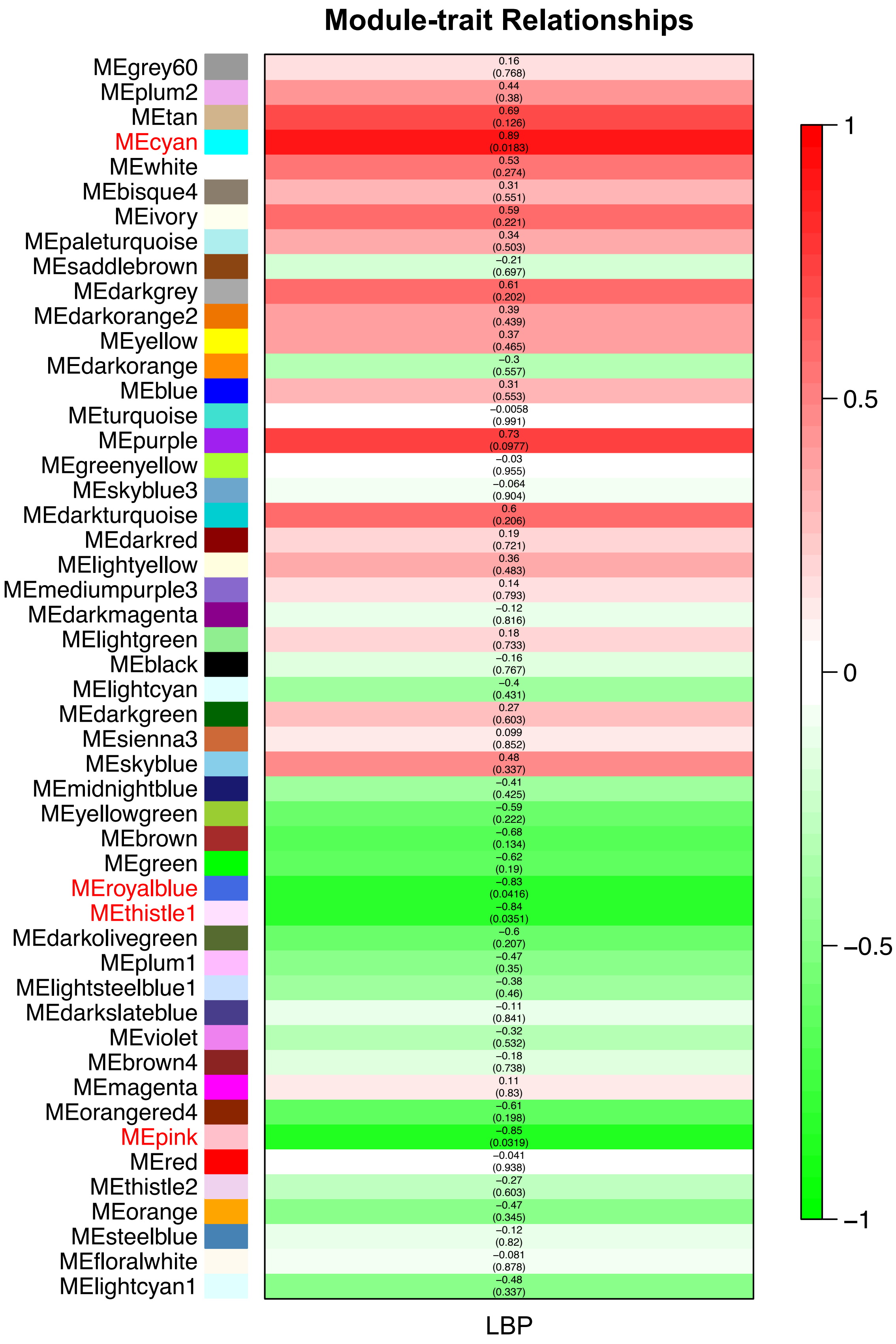

Supplementary Figure 11

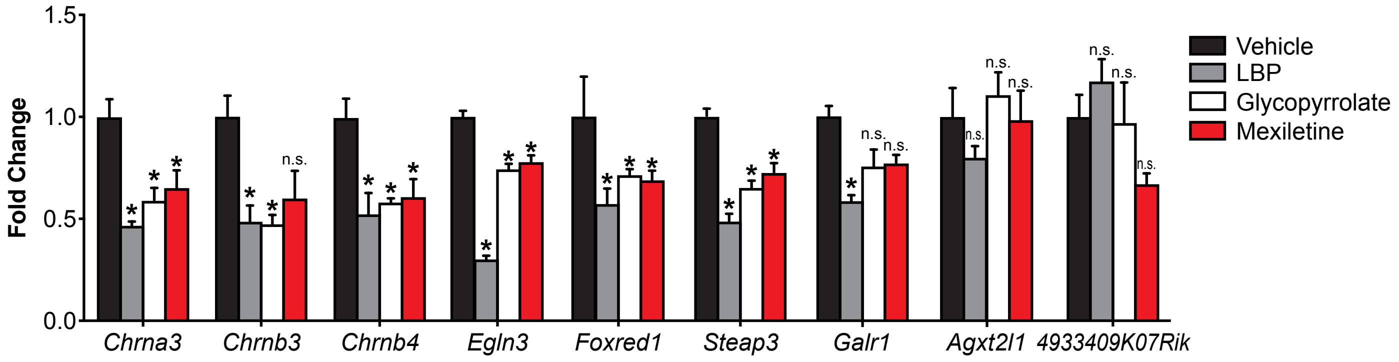

Supplementary Figure 12

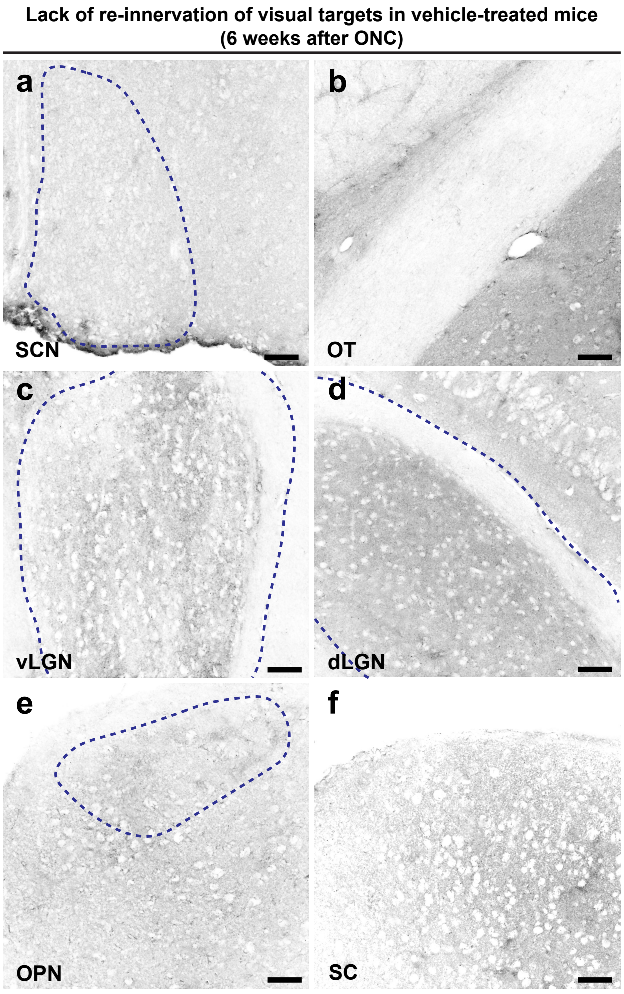

Supplementary Figure 13

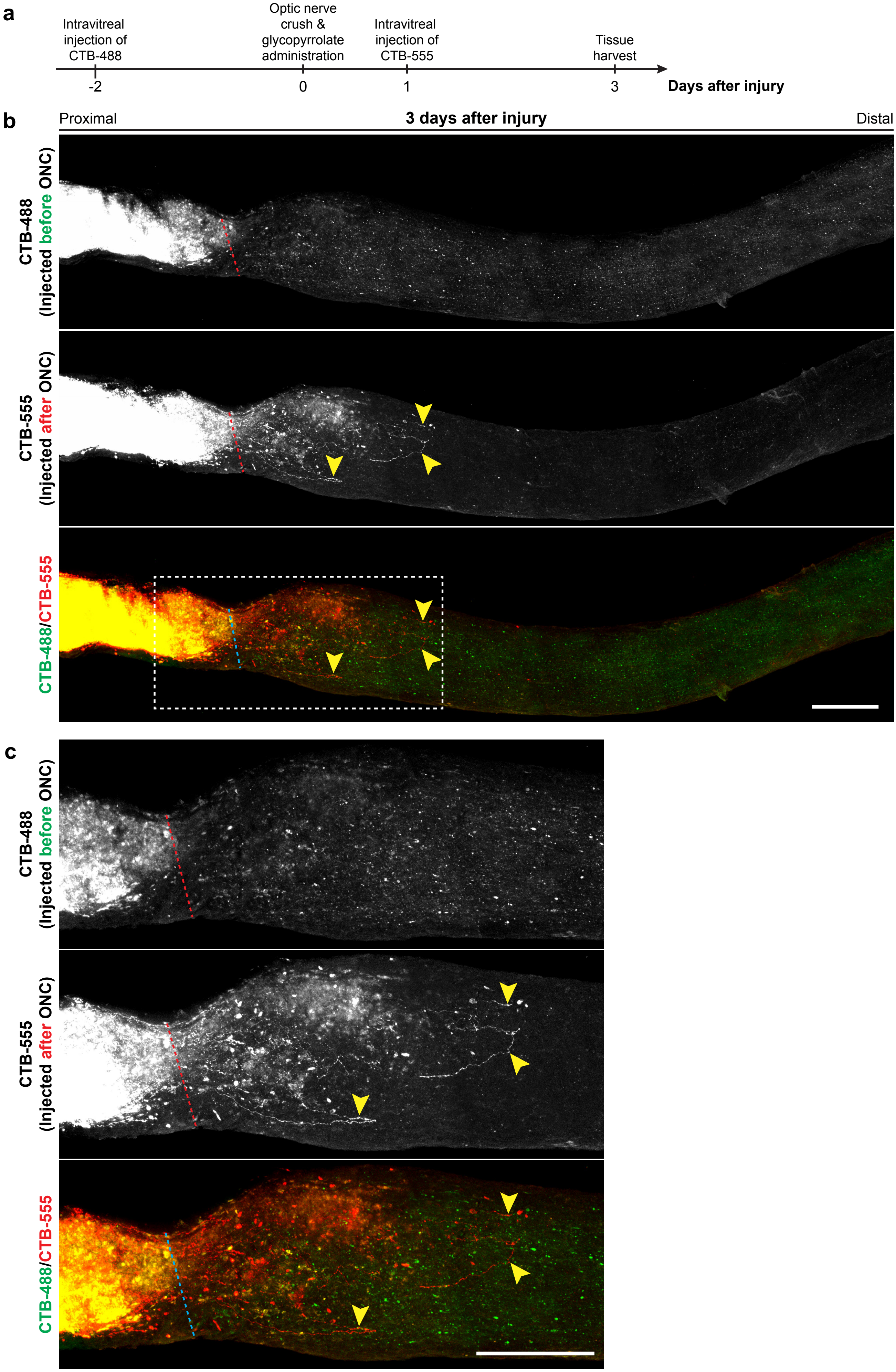

Supplementary Table 1

Gene name	log2 fold change after LBP treatment	*P* value
Gm3893	2.55	5.69E-09
4933409K07Rik	1.24	1.23E-06
Agxt2l1	0.92	8.62E-06
Chrnb3	-0.74	4.64E-05
Chrna3	-0.80	5.29E-05
Chrnb4	-0.76	9.63E-05
Egln3	-1.04	1.05E-04
Foxred1	-0.68	1.15E-04
Steap3	-0.48	4.80E-04
Galr1	-0.60	5.15E-04

Supplementary Table 2

Gene Name	Forward/Reverse	Sequence
Tnf-α	Forward	ACCACGCTCTTCTGTCTACT
	Reverse	GTTTGTGAGTGTGAGGGTCTG
Il-1β	Forward	GAGGACATGAGCACCTTCTTT
	Reverse	GCCTGTAGTGCAGTTGTCTAA
Il-6	Forward	CCAGTTCCTTCTTGGGACTG
	Reverse	CAGGTCTGTTGGGAGTGGTATCC
Cd86	Forward	GACCGTTGTGTGTGTTCTGG
	Reverse	GATGAGCAGCATCACAAGGA
Nos2	Forward	CCCTTCCGAAGTTTCTGGCAGCAGCG
	Reverse	GGCTGTCAGAGCCTCGTGGCTTTGG
Il-4	Forward	CCAAACGTCCTCACAGCAAC
	Reverse	AGGCATCGAAAAGCCCGAA
Il-10	Forward	GCCTTATCGGAAATGATCCA
	Reverse	TCTCACCCAGGGAATTCAAA
Cd206	Forward	CAAGGAAGGTTGGCATTTGT
	Reverse	CCTTTCAGTCCTTTGCAAGC
Tgf-β	Forward	TGCGCTTGCAGAGATTAAAA
	Reverse	CGTCAAAAGACAGCCACTCA
Igf-1	Forward	GCGATGGGGAAAATCAGCAG
	Reverse	CGCCAGGTAGAAGAGGTGTG
Ccl2	Forward	ACCTGCTGCTACTCATTCACC
	Reverse	GAGCTTGGTGACAAAAACTACA
Ccl3	Forward	ACAGCCGGAAGATTCCACG
	Reverse	TCTCTTAGTCAGGAAAATGACACC
Ccl4	Forward	CCCACTTCCTGCTGTTTCTCT
	Reverse	CCTCTTTTGGTCAGGAATACCA
Ccl5	Forward	GAGACATCCGTTCCCCCTAC
	Reverse	TAGGTCGGAACTGACCCTTG
Chrna3	Forward	CAGTGCCAACCTCACAAGAA
	Reverse	CCAGGATGAAAACCCAGAGA
Chrnb3	Forward	TCCAGGGAAAAAGAAGCAGA
	Reverse	AGAGGAAGATGCGGTCAAGA
Chrnb4	Forward	GGAGGTTCCGGCAAGATCTA
	Reverse	CACAATCACGAACACCCACA
Egln3	Forward	TGCTGAAGAAAGGGCAGAAG
	Reverse	CCGGCAAGAAAACATGAAGT
Foxred1	Forward	GCTCTAAGGCCATGTTTCGC
	Reverse	GACCAAGAATCCCACCTCCT
Steap3	Forward	AGGCCTTCAACGTCATCTCT
	Reverse	TATGGCTTCTACCTCCCTCG
Galr1	Forward	TCGTCTTTGGGTACCTTCTGC
	Reverse	TACTACAACGACCACCAGGAC
Agxt2l1	Forward	TGAGAAAGGCGAACGGTACT
	Reverse	GCTTGGCAAACTCAATGATG
4933409K07Rik	Forward	TCCCGAGAAGAAGCTGAGAA
	Reverse	ACCCTGGTTCAGTCCCAAAA
Gapdh	Forward	CATGGCCTTCCGTGTTCCTA
	Reverse	CCTGCTTCACCACCTTCTTGAT
